# MiR-4270 acts as a tumor suppressor by directly targeting Bcl-xL in human osteosarcoma cells

**DOI:** 10.3389/fonc.2023.1220459

**Published:** 2023-08-31

**Authors:** Clément Veys, Flavie Boulouard, Abderrahim Benmoussa, Manon Jammes, Emilie Brotin, Françoise Rédini, Laurent Poulain, Nicolas Gruchy, Christophe Denoyelle, Florence Legendre, Philippe Galera

**Affiliations:** ^1^ Normandie Univ., UNICAEN, BIOTARGEN, Caen, France; ^2^ Department of Genetics, Normandy Center for Genomic and Personalized Medicine, Caen University Hospital, Caen, France; ^3^ Research Center of the UHC Sainte-Justine and Department of Nutrition, Université de Montréal, Montréal, QC, Canada; ^4^ Normandie Univ., UNICAEN, Federative Structure Normandie Oncology, US Platon, ImpedanCELL Platform, Caen, France; ^5^ Normandie Univ., UNICAEN, INSERM U1086 ANTICIPE, Biology and Innovative Therapeutics for Ovarian Cancer (BioTICLA), Caen, France; ^6^ UNICANCER, Comprehensive Cancer Center F. Baclesse, Caen, France; ^7^ UMR 1238 Phy-Os “Bone Sarcomas and Remodeling of Calcified Tissues”, INSERM, Nantes University, Nantes, France

**Keywords:** osteosarcoma, chondrosarcoma, miR-4270, miR-342-5p, apoptosis, Bcl-XL

## Abstract

Chondrosarcomas and osteosarcomas are malignant bone tumors with a poor prognosis when unresectable or metastasized. Moreover, radiotherapy and chemotherapy could be ineffective. MiRNAs represent an alternative therapeutic approach. Based on high-throughput functional screening, we identified four miRNAs with a potential antiproliferative effect on SW1353 chondrosarcoma cells. Individual functional validations were then performed in SW1353 cells, as well as in three osteosarcoma cell lines. The antiproliferative and cytotoxic effects of miRNAs were evaluated in comparison with a positive control, miR-342-5p. The cytotoxic effect of four selected miRNAs was not confirmed on SW1353 cells, but we unambiguously revealed that miR-4270 had a potent cytotoxic effect on HOS and MG-63 osteosarcoma cell lines, but not on SaOS-2 cell line. Furthermore, like miR-342-5p, miR-4270 induced apoptosis in these two cell lines. In addition, we provided the first report of Bcl-xL as a direct target of miR-4270. MiR-4270 also decreased the expression of the anti-apoptotic protein Mcl-1, and increased the expression of the pro-apoptotic protein Bak. Our findings demonstrated that miR-4270 has tumor suppressive activity in osteosarcoma cells, particularly through Bcl-xL downregulation.

## 1 Introduction

Bone sarcomas are relatively rare tumors. Among the various bone sarcomas listed by the World Health Organization (WHO), the two main ones are osteosarcomas and chondrosarcomas. Osteosarcoma is the most common type of bone sarcoma (1, 2). This aggressive cancer mainly affects children and young adults, and, in the great majority of cases, it develops on the long bones of the limbs: the lower end of the femur, the upper end of the tibia or the upper end of the humerus (3). The tumor cells characteristically synthesize an osteoid matrix. Chondrosarcoma is the second most common bone sarcoma, affecting adults after the age of 40 years. It preferentially develops on flat bones (pelvis, scapula, rib, spine) and long bones (femur, tibia, humerus) (4, 5). This tumor is characterized by the formation of an extracellular cartilage matrix in a hypoxic environment (6).

In current treatments, osteosarcomas are the most curable sarcomas with a good prognosis for long-term survival. Neoadjuvant chemotherapy, including cisplatin, doxorubicin and methotrexate, coupled with surgery is the preferred therapy for this type of tumor (7, 8). However, for people with unresectable metastases or older people, prognosis is poor (9). For chondrosarcomas, only surgery is efficient, because chondrosarcomas are resistant to chemotherapy and radiotherapy (10). Overall, new therapeutic strategies are absolutely needed for the treatment of both types of bone tumors.

MicroRNAs (miRNAs) are small non-coding RNAs of about 20-25 nucleotides. They are post-transcriptional regulators of several hundred different mRNAs. They function as inhibitors of translation or they favor the degradation of the mRNAs when they anneal to mRNAs through complementary base pairing (11). In recent years, miRNAs have emerged as key regulators within complex networks of targets that have not yet been fully elucidated (12, 13). MiRNAs play an essential role in many physiological processes, but also in many pathological conditions, particularly in the progression of cancers, including bone sarcomas (14–16). Various miRNAs hold promise for use as diagnostic or prognostic biomarkers, and may also lead to the discovery of new therapeutic targets in bone sarcomas (14, 17–19). Many metabolic pathways regulated by miRNAs, such as survival, growth, angiogenesis or chemosensitivity, have been identified in bone sarcomas. However, few studies have focused on the effects of miRNAs directly targeting anti-apoptotic members of the Bcl-2 family in bone sarcomas. Several studies have reported the promising therapeutic potential of targeting these proteins for the treatment of bone sarcomas. The overexpression of Bcl-2 and Bcl-xL is indeed often associated with a poor prognosis and are partly responsible for the chemoresistance of tumor cells (20–24). One study has shown that pharmacological inhibition of Bcl-xL improves the sensitivity of osteosarcoma to doxorubicin (25). Furthermore, we previously showed that the direct inhibition of Bcl-xL by miR-491-5p and miR-342-5p induces apoptosis on osteosarcoma and chondrosarcoma cell lines (26, 27). MiR-342-5p also directly inhibited the expression of Bcl-2 in some tested cell lines.

In this study, we continued our previous high-throughput miRNA screening of 1200 miRNAs mimics on the SW1353 chondrosarcoma cell line (26). Using miRNA target prediction, we selected four miRNAs inducing a potential cytotoxic effect and which may potentially target *BCL2*, *BCL2L1* or *MCL1* mRNAs. We subsequently performed an individual and functional study with miR-16-1-3p, miR-646, miR-3667-3p and miR-4270. MiR-342-5p was used as a positive control because its tumor suppressive effects have already been validated on chondrosarcoma and osteosarcoma cells (26, 27). For the SW1353 chondrosarcoma cell line, the potential cytotoxic and chemosensitizing effects of miRNAs were studied in normoxia and hypoxia, because hypoxia is representative of the *in situ* physiopathological micro-environment of the tumor (28). Unfortunately, none of the newly selected miRNAs caused cytotoxic effects on SW1353 cells in the functional study. Because of their cytotoxic potential, we decided to test them on osteosarcoma cell lines. By contrast, miR-4270 had cytotoxic effects and induced cell death by apoptosis on two of the three osteosarcoma cell lines, suggesting a specific effect depending on cell type. Using western-blots and luciferase reporter assays, we demonstrated that miR-4270 directly targets *BCL2L1* mRNA (*Bcl-xL* gene). Moreover, miR-4270 decreased the expression of Mcl-1 at the protein level, whereas it increased that of Bak. These results suggest that miR-4270 can act as a tumor suppressor in osteosarcoma cells.

## 2 Materials and methods

### 2.1 Cell culture

The chondrosarcoma cell line SW1353 (ATCC® HTB-94) was cultured in high glucose-DMEM (HG-DMEM, Biowest) supplemented with 10% fetal calf serum (FCS, Eurobio Scientific, Courtaboeuf, France), 3 µg/mL ciprofloxacin (Sigma-Aldrich, Saint-Louis, MO, USA) and 0.5 µg/mL amphotericin (Eurobio Scientific). SW1353 cells were treated under normoxia (21% O_2_) and hypoxia (3% O_2_) in a sealed chamber (29).

The osteosarcoma cell line HOS (ATCC® CRL-1543™) was cultured in HG-DMEM supplemented with 10% FCS, and a cocktail of 100 IU/ml penicillin, 100 µg/ml streptomycin and 0.25 µg/ml amphotericin (Eurobio Scientific, Courtaboeuf, France). The osteosarcoma cell line MG-63 (ATCC® CRL-1427™) was cultured in MEM (Biowest, Nuaillé, France) supplemented with 10% FCS, and a cocktail of 100 IU/ml penicillin and 100 µg/ml streptomycin (Eurobio Scientific, Courtaboeuf, France). The osteosarcoma cell line SaOS-2 (ATCC® HTB-85™) was cultured in DMEM : HAM’s F12 (Biowest) supplemented with 5% FCS, 2 mM L-glutamine (Eurobio Scientific, Courtaboeuf, France), and a mixture of 100 U/ml penicillin and 100 µg/ml streptomycin (Eurobio Scientific, Courtaboeuf, France). The treatments of osteosarcoma cells were performed only under normoxia.

The human articular chondrocytes (HACs) were obtained with appropriate ethical approval, they were prepared from macroscopically healthy zones of femoral heads obtained from patients undergoing joint arthroplasty, as previously described (26). The study was performed in full accordance with local ethics committee guidelines and all the cartilage samples were collected after written and informed consent of the donors according to French legislation. All the experimental protocols were approved by the Agence de la Biomédecine (research protocol n° PFS21-025) and French Ministry of Higher Education and Research (Ethics Committee for Research on Human Samples CODECOH: DC 2014–2325).

### 2.2 Drugs and miRNAs

Sublethal doses of CDDP (Merck, Santé SAS, Lyon, France) were used during the transfection of miRNA as follows: 1 µg/ml (3.3 µM) for SW1353 and 0.5 µg/ml (1.65 µM) for HOS, MG-63 and SaOS-2 cells. MiRNA-Control (miR-Ctrl, MIMAT0000039), hsa-miR-342-5p (miR-342, MIMAT0004694), hsa-miR-16-1-3p (miR-16-1, MIMAT0004489), hsa-miR-646 (miR-646, MIMAT0003316), hsa-miR-3667-3p (miR-3667, MIMAT0018090), hsa-miR-4270 (miR-4270, MIMAT0016900) hsa-miRNA-342-5p-hairpin inhibitor (anti-miR-342, IH-301083-02), hsa-miR-4270-hairpin inhibitor (anti-miR-4270, IH-301835-01) and miRNA hairpin inhibitor Negative Control (anti-miR-ctrl, IN-001005-01) were purchased from Dharmacon (Horizon Discovery, Cambridge, UK).

### 2.3 miRNA mimic screening

SW1353 cells were plated in 96-well E-plates onto a xCELLigence real-Time Cell Analysis (RTCA, ACEA, Ozyme, Saint Quentin-en-Yvelines, France) under normoxia as previously described (26). Briefly, cells were transfected in triplicate 24 h after seeding with 20 nM miRNAs using Interferin™ (Polyplus-Transfection, Strasbourg, France). They were treated with or without CDDP 24 h post-transfection. Impedance of each well was continuously measured and expressed as a Cell index (CI) value ± SD (with the RTCA 2.1.0 software. Morphological analyses were done with the Cellavista Imaging System (Roche, Basel) at the end of the experiment (120 h after seeding) as previously described (26).

### 2.4 Transfection and CDDP treatment

Growing chondrosarcoma and osteosarcoma cells were seeded at 1x10^4^ cells/cm^2^ per 25 cm^2^ flask. Cell lines were transfected 24 h after seeding with 20 nM of miRNA mimics using INTERFERin™ (Polyplus-Transfection) as described previously (26, 27). An additional CDDP treatment was carried out after 48 h with each sublethal dose of CDDP as described previously (26, 27).

### 2.5 XTT assay

SW1353 cells and osteosarcoma cell lines were seeded in a 96-well plate at a density of 3.5x10^3^ cells/well and 3.3x10^3^ cells/well respectively in triplicate. Cells were transfected with 20 nM miRNA 24 h later with or without an additional CDDP treatment 48 h post-transfection. Cell metabolism activity was assessed 72 h post-transfection using the XTT assay (Roche, Basel, Switzerland) as previously described (26, 27). Measurements of optical density (OD) were made with a microplate reader (Spark 10M, Tecan Lyon, France).

### 2.6 Bioluminescence cytotoxicity assay

The toxilight cytotoxicity assay kit (Interchim, Montluçon, France) was also used to evaluate miRNA-induced cytotoxicity 72 h post-transfection. Adenylate Kinase released was measured in the supernatant, as previously described (26). The bioluminescence was measured with a microplate reader (Spark 10M, Tecan Lyon, France) and expressed as relative luciferase units (RLU).

### 2.7 Cell cycle analysis by DNA content

DNA content after staining of trypsinized cells was performed by nuclear staining with propidium iodide (Sigma-Aldrich, Saint-Louis, MO, USA) using a Cytoflex S Flow Cytometer (Beckman Coulter France SAS, Paris, France) as previously described (26).

### 2.8 Nuclear morphology

Morphological characterization of detached cells and trypsinized cells by nuclear staining with DAPI (4’,6-diamidino-2-phenylindole, dilactate) (Santa Cruz Biotechnology, Dallas, TX, USA) was carried out as previously described (26).

### 2.9 Western-blots

Protein levels of both detached and adherent cells were analyzed by immunoblotting with PARP (9542), Bcl-xL (2764) and Bak (3814) antibodies from Cell Signaling Technology (Ozyme, Saint-Quentin-en-Yvelines, France), with Mcl-1 (S-19), GAPDH (FL-335) and β-Tubulin (D-10) antibodies from Santa Cruz Biotechnology (Dallas, TX, USA), and with the Bcl-2 (M0887) antibody from DAKO (Agilent, Santa Clara, CA, USA) as previously described (26, 27). Each immunoblot is representative of at least three distinct experiments. Protein expression was estimated by quantifying the density of immunoblots bands adjusted to GAPDH or β-Tubulin (ImageLab® software).

### 2.10 miRNA target reporter assay

HOS cells were co-transfected for 48 hours using jetOPTIMUS**™** DNA transfection reagent (Polyplus-Transfection, Strasbourg, France) with 20 nM miRNA mimic or miRNA hairpin inhibitor and with BCL2-3’UTR vector (217HmiT016211a; 2761 bp), BCL2L1-3’UTR vector (217HmiT108616-MT05; 1489 bp) or MCL1-3’UTR vector (217HmiT127389-MT05; 2839 bp) (1 ng/µL; GeneCopoeia, Rockville, MD, USA) as previously described (27). These vectors contain the miR-342-5p and miR-4270 3’UTR sequences downstream a Gaussia luciferase (GLUC) reporter gene and a secreted alkaline phosphatase (SEAP) reporter gene. SEAP and GLUC luciferase activities were assayed in triplicate with the secrete-pair dual luminescence assay kit (GeneCopoeia, Rockville, MD, USA). Reporter activities were measured with a luminescence microplate reader (Spark 10M, Tecan) and expressed as relative luciferase units (RLU) corresponding to the GLUC : SEAP ratio.

### 2.11 Caspase-3/7 activity

Caspase-3/7 activity of HOS, MG-63 and SaOS-2 cells was assessed using the Incucyte® Caspase-3/7 Green Apoptosis Assay Reagent (Fisher Scientifics SAS, Illkirch, France) in the Incucyte® S3 acquiring images (Essen BioScience, Ltd., Royston, UK) as previously described (26, 27). Briefly, cells were monitored after transfection of 20 nM miRNA during 72 h. Each experiment was done in triplicate.

### 2.12 Statistical analysis

All experiments were repeated at least three times. Values are presented as means ± SD. Statistical analyses were performed using one-way ANOVA corrected for multiple comparisons using Dunnett’s test, or two-way ANOVA corrected for multiple comparisons using Sidak’s test. Alternatively, for representative experiments performed in triplicate, statistical analyses were done using a two-tailed unpaired Student’s *t*-test with Welch’s correction. Analyzes were done using GraphPad Prism 7 software (San Diego, CA, USA): NS, P > 0.05; *, p <0.05; **, p < 0.01; ***, p < 0.001).

## 3 Results

### 3.1 Identification of antiproliferative miRNAs using high-throughput screening on the SW1353 chondrosarcoma cell line

**Figure 1:**
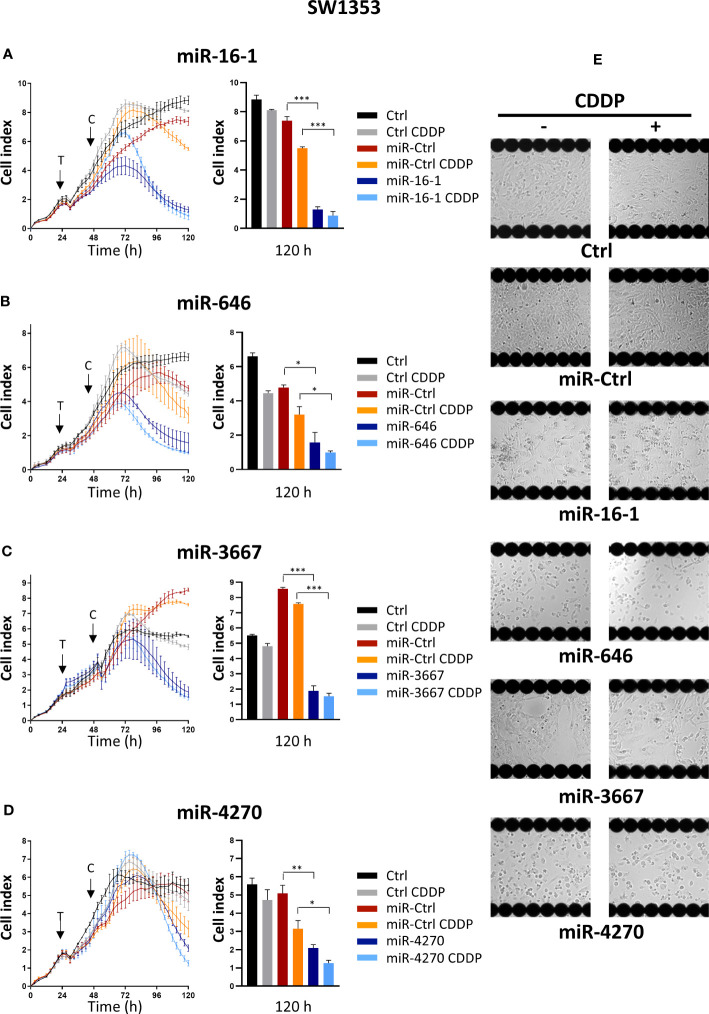
High-throughput screening of miRNAs on SW1353 chondrosarcoma cells. SW1353 cells were grown for 24 h before transfection (arrow labeled with a T) with miR-Ctrl, miR-16-1-3p, miR-646, miR-3667-3p or miR-4270. The cells were additionally treated with CDDP (1 µg/ml) 24 h post-transfection (arrow labeled with a C). **(A–D)** Real-time growth curves monitoring was performed with the xCELLigence System. The measurement of Cell index was monitored every 3 h for 120 h. Analysis of one experiment per miRNA, with mean Cell index ± SD, is shown for 120 h (left panels) and at 120 h after seeding (right panels). The significance of the results was evaluated using an unpaired Student’s *t*-test. **(E)** Endpoint morphological analysis of the cells was done 120 h after seeding with the Cellavista Imaging system. Ctrl, control; CDDP, cisplatin. *: p<0.05, **: p<0.01, ***: p<0.001.

We performed a real-time high-throughput screening of 1200 miRNA mimics on the SW1353 cells, as described previously (26). An additional cisplatin (CDDP) treatment was also realized 24 h after transfection of miRNAs to study their chemosensitizing effects (26). Biologicals effects of miRNAs were analyzed by following Cell Index (CI) in real-time using the xCELLigence System and by endpoint morphological observations of the cells using the Cellavista Imaging System. Finally, among all these miRNAs, we selected four novel miRNAs (miR-16-1-3p, miR-646, miR-3667-3p and miR-4270) with potential effectiveness on cell proliferation/attachment (**Figure 1**).

MiR-16-1-3p decreased the cell index from approximately 60 h post-transfection until the end of the experiment (5.7-fold decrease compared with miR-Ctrl, 120 h after seeding, i.e. 96 h after transfection, p<0.001, **Figure 1A**). In the presence of a sublethal dose of CDDP, the decrease in the cell index with miR-16-1-3p was delayed to the same degree as without CDDP 120 h after plating the cells (p<0.001, **Figure 1A**). Microscopic observations of the cells 120 h after seeding confirmed a decrease in proliferation with miR-16-1-3p, with or without CDDP (**Figure 1**). MiR-16-1-3p therefore showed antiproliferative effects on SW1353 cells without chemosensitizing effects.

MiR-646 also decreased the cell index from 48 h post-transfection until the end of the experiment (3-fold decrease relative to miR-Ctrl, 120 h after seeding, p<0.05, **Figure 1B**). When miR-646 was combined with CDDP, the decrease was 3.2-fold (relative to miR-Ctrl + CDDP, 120 h after seeding, p<0.05, **Figure 1B**), suggesting no significant sensitization to CDDP. Moreover, miR-646- and miR-646/CDDP-treated cells showed a similar large reduction in confluence compared with the respective miR-Ctrl-treated cells (**Figure 1**). Therefore, miR-646 had only antiproliferative effects on SW1353 cells.

Similarly, the transfection of miR-3667-3p resulted in a decrease in the cell index by 4.7-fold at the end of the experiment with or without CDDP (p<0.001, relative to the respective miR-Ctrl treatments, **Figure 1C**). Cell attachment and cell density also decreased compared with respective miR-Ctrl (**Figure 1**). MiR-3667-3p appeared to have only antiproliferative effects on SW1353 cells.

Finally, miR-4270 also reduced the cell index, but its effects were delayed compared with the other three miRNAs, with an effect observed only from approximately 72 h post-transfection (**Figure 1**). At the end of the experiment, miR-4270 decreased the cell index by 2.5-fold with or without CDDP (p<0.01 compared with miR-Ctrl and p<0.05 compared with miR-Ctrl/CDDP, at 120 h after seeding). Cell confluency also decreased by at least 50% with or without CDDP, suggesting no chemosensitizing effect of this miRNA (**Figure 1**). Therefore, miR-4270 had only antiproliferative effects on SW1353 chondrosarcoma cells.

### 3.2 Individual functional analysis of miRNAs fails to confirm induction of cell death in the SW1353 chondrosarcoma cell line

Next, we carried out a functional analysis with these four miRNAs and with miR-342-5p, used as a positive control, at 72 h post-transfection on SW1353 cells cultured under normoxia and hypoxia. We also investigated the potential chemosensitizing effects of the miRNAs with a sublethal exposure to CDDP 48 h after their transfection.

The studies of SW1353 viability and proliferation and of the cytotoxicity of miRNAs showed the best antiproliferative and cytotoxic effects for miR-342-5p and no relevant effect of other miRNAs, except with miR-16-1-3p and miR-646 under hypoxia with CDDP, thereby revealing a possible chemosensitizing action (**Figure S1A**). Nevertheless, this effect was not further confirmed, because both of these latter miRNAs failed to induce cytotoxicity on SW1353 cells in hypoxia, with or without CDDP (**Figure S1B**), unlike miR-342-5p as previously described (26).

Regarding cell cycle progression, no cell cycle blockade was observed with miRNAs using flow cytometry (**Figure S2**). None of miRNAs induced a cell cycle blockade in SW1353 cells. On the contrary, the sublethal dose of CDDP induced a cell cycle blockade in the S and G2/M phases. Only miR-342-5p increased the number of cells in the sub-G1 phase in all four conditions of culture (approximately of 3-fold, p<0.01 or p<0.05 relative to the respective miR-Ctrl treatments). The proportions of the cells in the sub-G1 phase for miR-Ctrl and miR-342-5p respectively were 6.8% and 21.9% without CCDP under normoxia; 5.5% and 14% with CDDP under normoxia; 6.6% and 20.5% without CDDP under hypoxia; 4.4% and 14.4% with CDDP under hypoxia.

Microscopic observations at 72 h post-transfection further corroborated that only miR-342-5p induced cell death. Indeed, miR342-5p decreased the confluency and increased the number of apoptotic debris or cellular debris (**Figure S3**). MiR-646 significantly increased the percentage of sub-G1 events by 2-fold (p<0.05 relative to miR-Ctrl/CDDP treated cells) only under normoxia in the presence of CDDP (**Figure S2**). MiRNAs did not increase the sub-G1 peaks in the presence of CDDP, suggesting no chemosensitizing effect for all miRNAs tested.

Overall, we concluded that the antiproliferative effects of these four selected miRNAs, observed in the high-throughput screening were not due to an induction of cell death in SW1353 cells.

### 3.3 Functional analysis of miRNAs reveals antiproliferative effects of miR-342-5p, miR-16-1-3, miR-646 and miR-4270 on osteosarcoma cell lines

**Figure 2:**
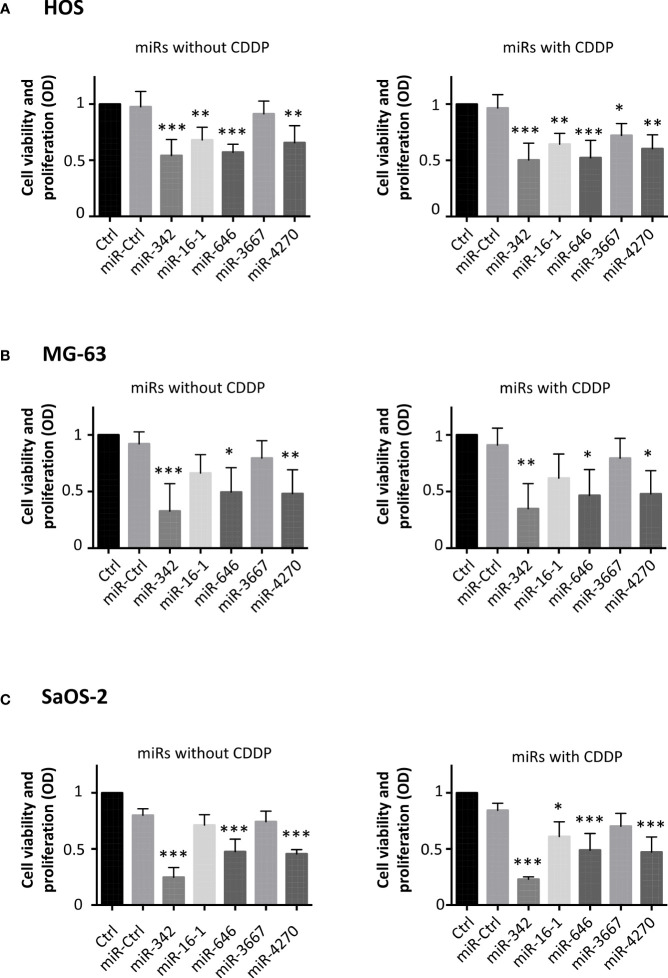
Effects of miRNAs on cell viability and proliferation on osteosarcoma cell lines. **(A)** HOS, **(B)** MG-63 and **(C)** SaOS-2 cells were transfected with miRNAs 24 h after seeding and treated with CDDP (0.5 µg/ml) 48 h post-transfection for 24 **(h)** Analyses were then carried out 72 h post-transfection. Cell viability and proliferation were evaluated and expressed as the mean OD ± SD of four independent experiments for each cell line. The significance of the results between miR-Ctrl and miRNA-treated cells was evaluated using one-way ANOVA (*: p<0.05, **: p<0.01, ***: p<0.001).

In parallel, we performed a functional analysis on three osteosarcoma cell lines at 72 h post-transfection. Cells were treated under normoxia in the presence or absence of a sublethal dose of CDDP 48 h after the transfection of miRNAs. First, cell viability and proliferation were evaluated using an XTT assay (**Figure 2**).

In HOS cells (**Figure 2**), all miRNAs (except miR-3667) significantly decreased the cell viability and proliferation compared with miR-Ctrl (by about 1.8-fold for miR-342-5p and miR-646, p<0.001; by about 1.5-fold for miR-16-1-3p and miR-4270, p<0.01). In the presence of CDDP, all miRNAs decreased the cell viability and proliferation. MiR-342-5p and miR-646 were the most effective miRNAs with the same decrease with or without CDDP (by about 1.8-fold (p<0.001)). MiR-3667-3p was the least effective with a weak decrease of only 1.3-fold in the presence of CDDP (p<0.05).

Regarding MG-63 cells, miR-342-5p, miR-646 and miR-4270 significantly decreased their viability and proliferation at 72 h post-transfection (**Figure 2**). MiR-342-5p decreased the cell viability and proliferation by approximately 3-fold in both culture conditions (p<0.001 relative to miR-Ctrl, and p<0.01 relative to miR-Ctrl/CDDP), whereas miR-646 and miR-4270 had lower magnitude effects with a decrease of approximately 2-fold (p<0.05 relative to respective miR-Ctrl).

MiR-342-5p, miR-646 and miR-4270 significantly decreased SaOS-2 viability and proliferation when used without CDDP (3.2-fold decrease for miR-342-5p and 1.7-fold for the two other miRNAs, p<0.001 relative to miR-Ctrl) (**Figure 2**). When miRNAs were associated with a sublethal dose of CDDP, all miRNAs (except miR-3667) significantly decreased the cell viability and proliferation (3.7-fold decrease for miR-342-5p, p<0.001; about 1.8-fold for miR-646 and miR-4270, p<0.001; 1.4-fold for miR-16-1-3p, p<0.05).

In summary, we observed recurrent antiproliferative effects of miR-342-5p, miR-16-1-3, miR-646 and miR-4270 on the three osteosarcoma cell lines.

### 3.4 Functional analysis of miRNAs reveals cytotoxic effects of miR-4270 on HOS and MG-63 osteosarcoma cell lines, but not on the SaOS-2 osteosarcoma cell line

**Figure 3:**
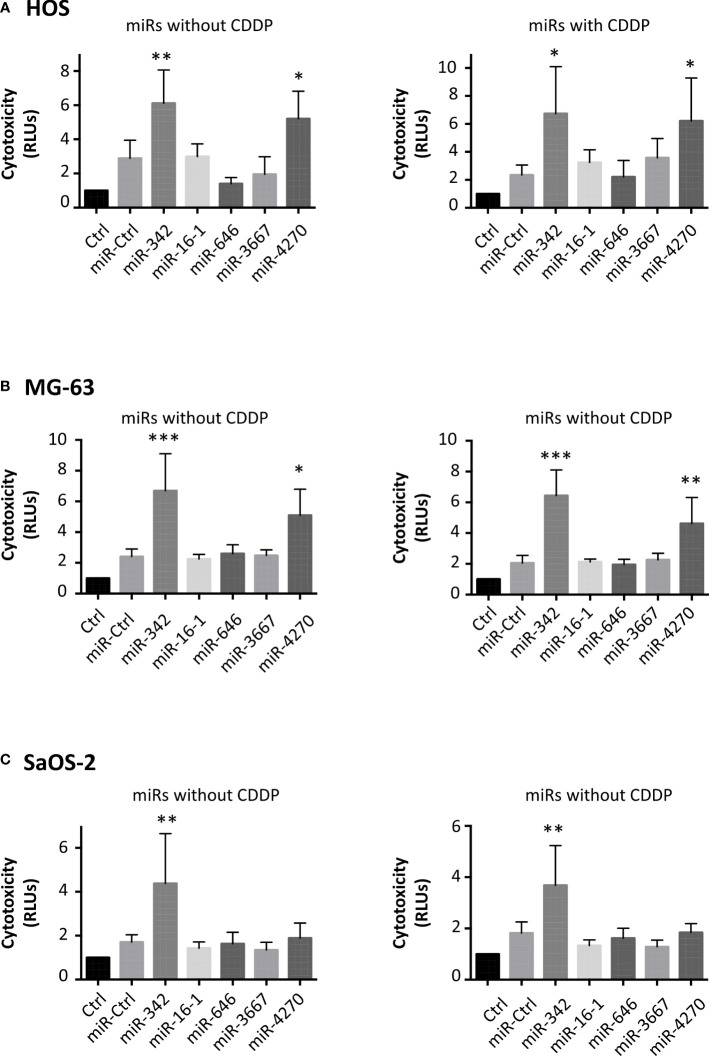
Cytotoxic effects of miRNAs on osteosarcoma cell lines. **(A)** HOS, **(B)** MG-63 and **(C)** SaOS-2 cells were transfected with miRNAs and treated with CDDP as described in **Figure 2**. The cytotoxicity of miRNAs was evaluated 72 h post-transfection as the mean RLU ± SD of four independent experiments for each cell line. The significance of the results between miR-Ctrl and miRNA-treated cells was evaluated using one-way ANOVA (*: p<0.05, **: p<0.01, ***: p<0.001).

We then analyzed the cytotoxic effects of these miRNAs (**Figure 3**). In addition to miR-342-5p, only miR-4270 significantly induced high cytotoxicity on HOS cells (**Figure 3**): 1.4-fold relative to miR-Ctrl treated cells (p<0.05), and 2.4-fold relative to miR-Ctrl/CDDP treated cells (p<0.05). In the same manner, only miR-4270 and miR-342-5p induced high cytotoxicity on MG-63 cells, by approximately 2-fold (with and without CDDP) *versus* approximately 3-fold with miR-342-5p (**Figure 3**). None of the four studied miRNAs induced cytotoxicity on SaOS-2 cells. Only the positive control miR-342-5p increased cytotoxicity by about 2-fold in the presence or absence of a sublethal dose of CDDP (**Figure 3**).

**Figure 4:**
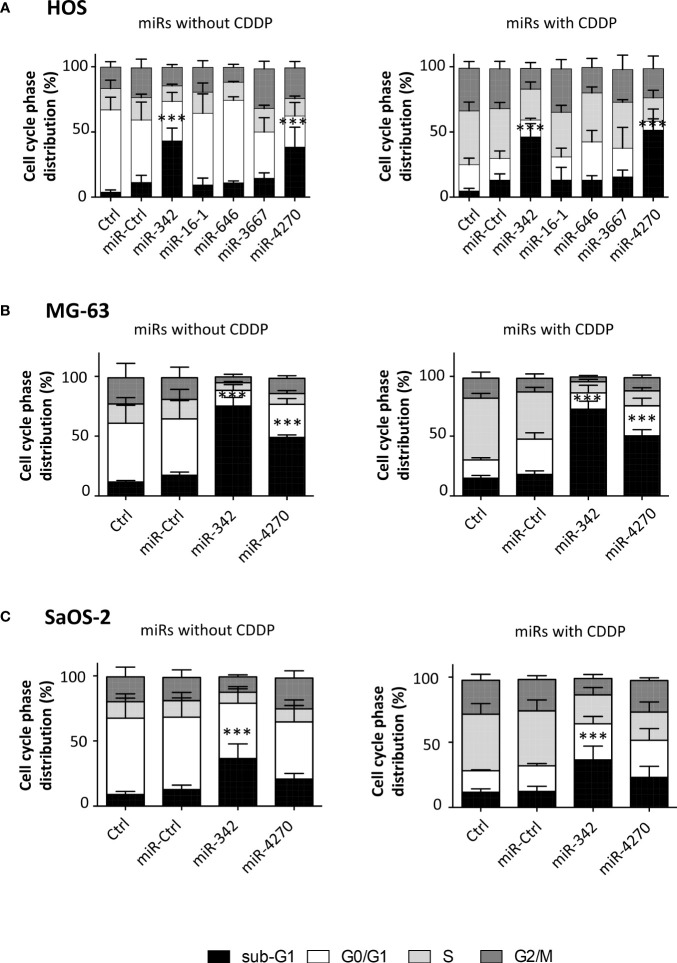
Analysis of cell cycle phase distribution after miRNA treatment on osteosarcoma cell lines. **(A)** HOS, **(B)** MG-63 and **(C)** SaOS-2 cells were transfected with miRNAs and treated with CDDP as described in **Figure 2**. The cell cycle phase distribution was evaluated 72 h post-transfection as the mean ± SD of four independent experiments for HOS and MG-63 cells, and for five independent experiments for SaOS-2 cells. The significance of the results between miR-Ctrl and miRNA-treated cells was evaluated using one-way ANOVA (***: p<0.001).

In the HOS cell line, the analysis of cell cycle cell progression using flow cytometry showed a significant enhancement in the number of sub-G1 events for only miR-342-5p and miR-4270 (**Figure 4**). Without CDDP, miR-342-5p significantly increased the number of sub-G1 events by 3.8-fold (p<0.001; 11.2% of sub-G1 events with miR-Ctrl and 43.1% with miR-342-5p) and miR-4270 increased it by 3.4-fold (p<0.001; 38.4% of sub-G1 events with miR-4270). CDDP increased the percentage of events in the S and G2/M phases at the expense of the G0/G1 phase. In the presence of CDDP, miR-342-5p increased cell death by 3.5-fold (p<0.001; 13% of sub-G1 events with miR-Ctrl and 46.2% with miR-342-5p) and miR-4270 by 3.9-fold (p<0.01; 51.4% of sub-G1 events with miR-4270). There was no significant difference in the induction of cell death between cells transfected with miRNAs alone or with miRNAs combined with CDDP treatment. The analysis of cell morphology at 72 h post-transfection showed a decrease in cell confluence and an increase in the amount of cellular debris as well as fragmented chromatin — signs typical of apoptotic cells — for both miRNAs with or without CDDP (**Figure S4**).

The analysis of the cell cycle distribution on MG63 cells revealed that the treatment with a sublethal dose of CDDP induced a blockage in the S phase only (**Figure 4**). MiR-4270 significantly increased the number of sub-G1 events by 2.8-fold for both culture conditions (p<0.001 relative to miR-Ctrl with or without CDDP) (**Figure 4**). In comparison, miR-342-5p increased cell death by about 4-fold for both conditions (p<0.001) (**Figure 4**), as previously described (27). For both miRNAs, morphological analysis of the cells at 72 h post-transfection showed a large increase in the amount of cellular debris and in the presence of apoptotic bodies compared with the miR-Ctrl condition with or without CDDP (**Figure S5**).

In the SaOS-2 cell line, we observed that CDDP increased the percentage of events in the S phase at the expense of the G0/G1 phase (**Figure 4**). Only miR-342-5p was able to significantly increase the number of sub-G1 events by 2.9-fold in the presence or absence of a sublethal dose of CDDP (p<0.001). MiR-4270 did not significantly modulate cell death in either culture conditions: 1.6-fold increase relative to miR-Ctrl and 1.9-fold relative to miR-Ctrl/CDDP. Morphological analysis of the cells showed a reduction in confluency for both miRNAs, but fewer apoptotic bodies with miR-4270 than with miR-342-5p (**Figure S6**).

Overall, these results suggest that, similarly to miR-342-5p, miR-4270 may be an apoptomiR in HOS and MG63 osteosarcoma cell lines, whereas it had only a significant antiproliferative effect on the SaOS-2 osteosarcoma cell line. MiR-4270 induced cell death to the same magnitude with or without CDDP, suggesting that it is not a CDDP chemosensitizer.

### 3.5 MiR-4270 activates the apoptosis pathway in the HOS and MG-63 osteosarcoma cell lines

**Figure 5:**
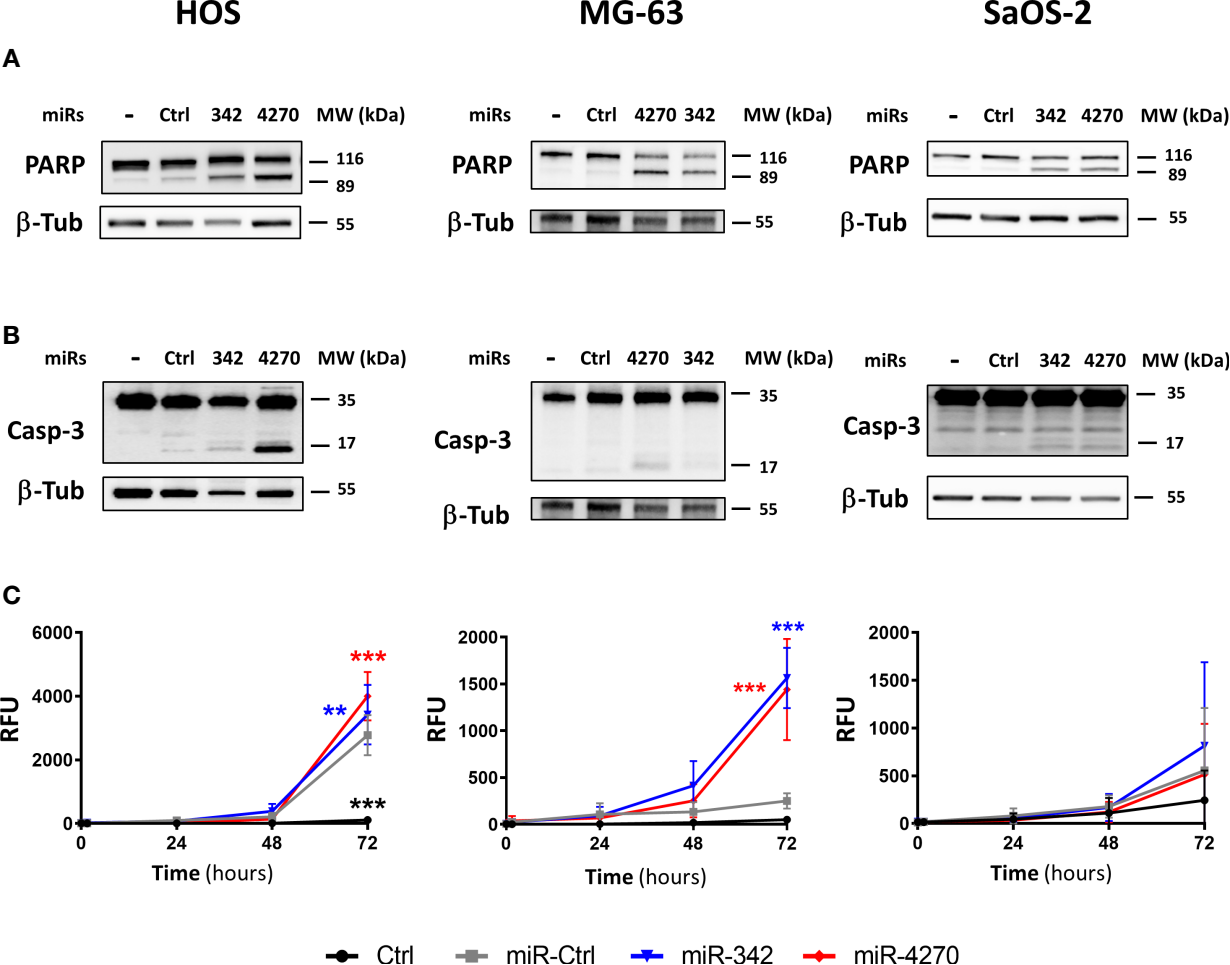
Analysis of the apoptotic effects of miR-342-5p and miR-4270 on osteosarcoma cell lines. HOS, MG-63 and SaOS-2 cells were transfected with miRNAs 24 h after seeding. In panels **(A, B)**, PARP and caspase-3 levels were analyzed 72 h post-transfection. Representative blots of three independent experiments are shown. **(C)** The caspase-3/7 activity was assessed for 72 **(h)** (mean RFU ± SD; n=5 for HOS and SaOS-2 cells; n=4 for MG-63 cells). The significance of the results between miR-Ctrl and miRNA-treated cells was assessed using two-way ANOVA (**: p<0.01, ***: p<0.001).

We then investigated the induction of apoptosis by miR-4270 alone in the three osteosarcoma cell lines 72 h post-transfection (**Figure 5**). We noted that miR-Ctrl induces cleavage of PARP (**Figure 5A**, **S7**) and increases caspase 3/7 activity in the HOS line (**Figure 5**). This cell line appears to be more sensitive to transfection as previously described (27). Similar to miR-342-5p, miR-4270 induced PARP cleavage in the three osteosarcoma cell lines (**Figure 5**). The effects were more relevant in the HOS and MG-63 cell lines than in the SaOS-2 cell line. A high amount of cleaved caspase-3 was detected with miR-4270 transfection, particularly in the HOS cell line, but this cleavage was almost absent in the SaOS-2 cell line for both miRNAs (**Figure 5**). In addition, miR-4270 significantly increased caspase-3/7 activity only in HOS and MG-63 cells (by 1.8-fold and by 5.7-fold relative to miR-Ctrl treated cells, respectively, p<0.001) (**Figure 5**). MiR-342-5p significantly induced caspase-3/7 activity, to the same magnitude as miR-4270, only in HOS and MG-63 cells as previously described (27)). Because the activation of caspases and the cleavage of PARP are characteristic hallmarks of apoptosis, these results suggest that miR-4270 unambiguously induces apoptosis in the HOS and MG-63 osteosarcoma cell lines, like miR-342-5p. This is in accordance with our previous results on cell death (**Figures 3**, **4**).

### 3.6 MiR-4270 modulates the expression of proteins of the Bcl-2 family in osteosarcoma cell lines

Bcl-xL, Bcl-2, Mcl-1 are all members of the anti-apoptotic Bcl-2 family and are among the predicted targets of miR-4270 and miR-342-5p. We therefore evaluated their protein expression to determine their involvement in the induction of apoptosis by this miRNA in the three osteosarcoma cell lines.

**Figure 6:**
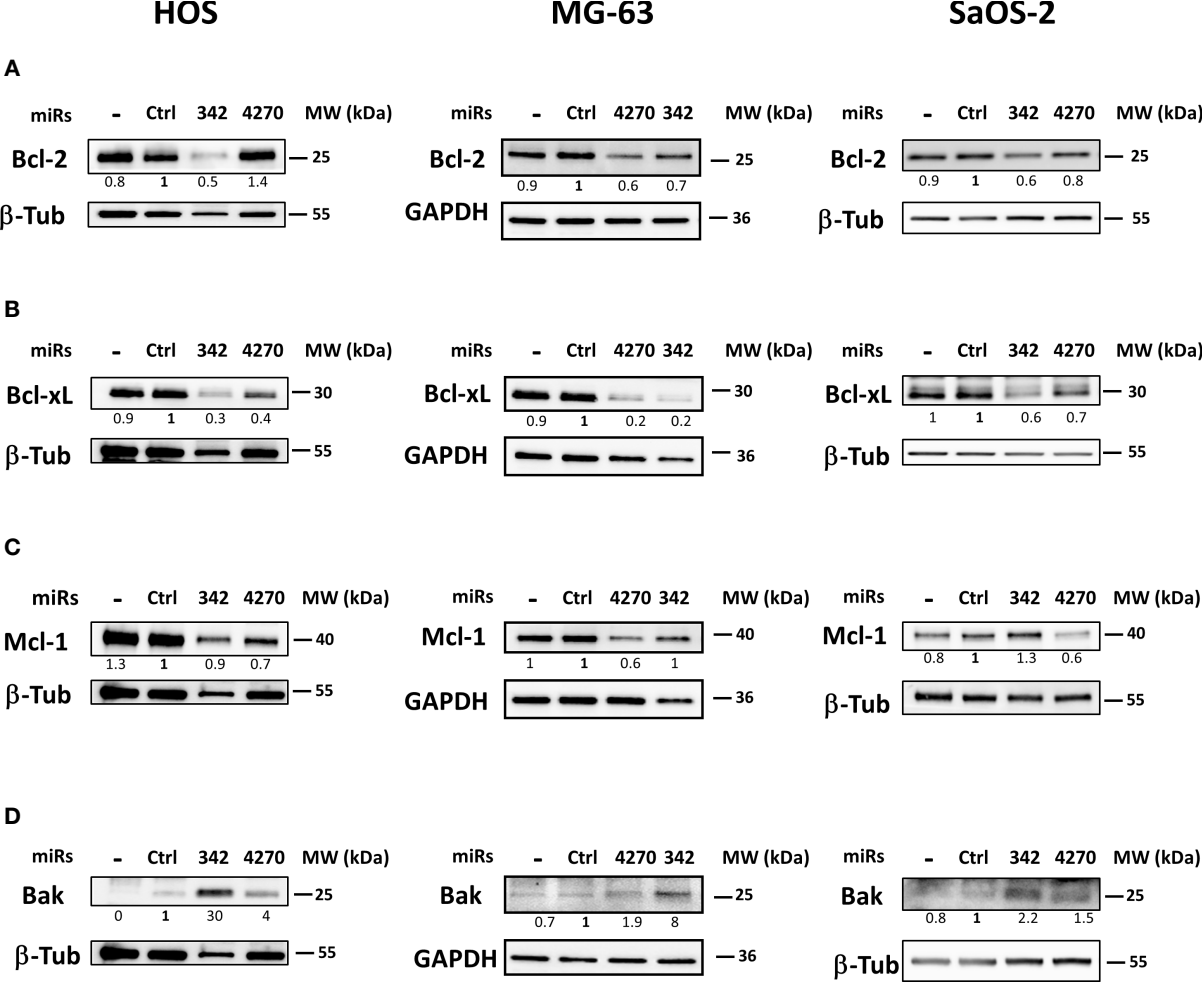
Effects of miR-342 and miR-4270 on the expression of members of the Bcl-2 family in osteosarcoma cell lines. In panels **(A–D)**, HOS, MG-63 and SaOS-2 cells were transfected with miRNAs 24 h after seeding. Bcl-2, Bcl-xL, Mcl-1 and Bak levels were analyzed 72 h post-transfection. Representative blots of three independent experiments are shown. Protein expressions are indicated at the bottom of the blots. They were normalized to β-tubulin or GAPDH and to the corresponding miR-Ctrl.

Bcl-2 protein expression did not decrease, but increased (by 1.4-fold relative to miR-Ctrl) with miR-4270 in HOS cells (**Figure 6**). By contrast, miR-342-5p decreased Bcl-2 protein expression by 2-fold in the HOS cell line. MiR-4270 decreased Bcl-2 protein expression by 1.7-fold and 1.2-fold, respectively, in MG-63 and SaOS-2 cells. The effects of miR-342-5p were comparable on both these two cell lines (1.4-fold decrease in MG-63 cells and 1.7-fold in SaOS-2 cells).MiR-4270 significantly decreased Bcl-xL protein expression in all three osteosarcoma cell lines (by 2.5-fold in HOS cells, 5-fold in MG-63 cells, and 1.4-fold in SaOS-2 cells) (**Figure 6**). We previously validated Bcl-xL as a direct target of miR-342-5p in the HOS cell line (27). Here, we confirmed its inhibitory effect on Bcl-xL protein expression in the three tested osteosarcoma cell lines with at least a 1.7-fold decrease.

*MCL-1* mRNA is a putative target for both miRNAs. MiR-342-5p had no relevant effect on the level of Mcl-1 protein expression (**Figure 6**). In all three osteosarcoma cell lines, miR-4270 decreased Mcl-1 protein expression at different levels: by 1.4-fold in HOS cells and by 1.7-fold in MG-63 and SaOS-2 cells.

We then investigated the expression of the pro-apoptotic protein Bak, involved in the intrinsic apoptosis pathway (**Figure 6**). This protein participates in mitochondrial outer membrane permeabilization and subsequent induction of cell death. Both miRNAs clearly induced Bak protein expression in the three osteosarcoma cell lines. MiR-342-5p had the strongest effect (from 2.2-fold to 30-fold induction with miR-342-5p but only from 1.5-fold to 4-fold induction with miR-4270).

In summary, miR-4270 mainly decreased the expression of the anti-apoptotic proteins Bcl-xL and Mcl-1, whereas miR-342-5p mainly decreased that of Bcl-xL and also Bcl-2 in osteosarcoma cells. Both miRNAs significantly increased the expression of the pro-apoptotic protein Bak.

### 3.7 Direct inhibition of BCL2L1 mRNA by miR-4270 in HOS osteosarcoma cell line

**Figure 7:**
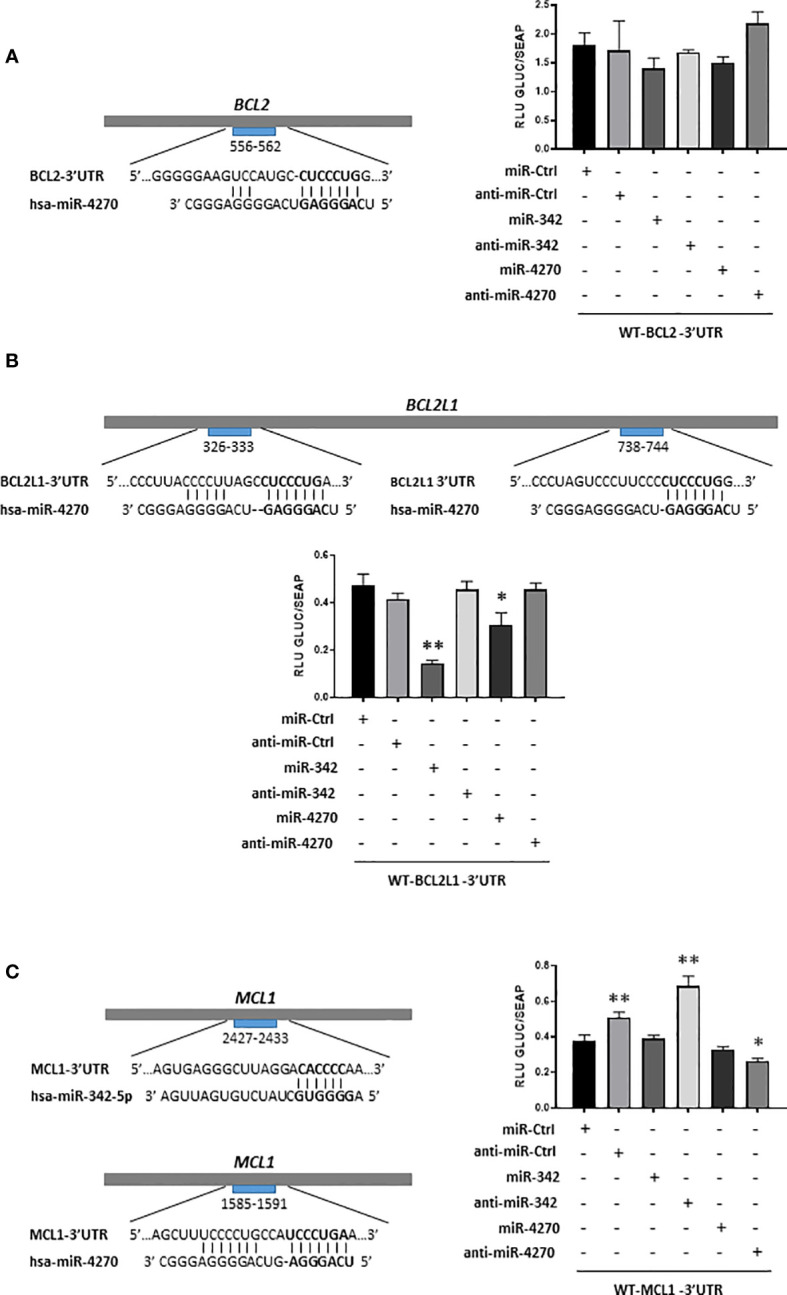
Analysis of inhibition of *BCL2*, *BCL2L1* and *MCL1* mRNAs by miR-342-5p and miR-4270 in HOS cells. Schematic representations of the 3’UTR sequences of *BCL2*, *BCL2L1* and *MCL1* are respectively shown in panels **(A–C)**, with predicted sites of hsa-miR-4270 or hsa-miR-342-5p. Reporter vectors of BCL2-3’UTR, BCL2L1-3’UTR and MCL1-3’UTR were transfected concomitantly with miR or anti-miR in HOS cells. Reporter assays were carried-out 48 h post-transfection. Gaussia luciferase (GLUC) activity was normalized to Secreted alkaline phosphatase (SEAP) activity. The data show representative experiments of at least three independent experiments (mean of RLU ± SD). The significance of the values between the different treatments and miR-Ctrl was evaluated using two-tailed Student’s *t*-test (*: p<0.05, **: p<0.01).

We then verified that the effect of miR-342-5p and miR-4270 on Bcl-2 (*BCL2* gene), Bcl-xL (*BCL2L1* gene) or Mcl-1 (*MCL1* gene) resulted from direct binding to their respective mRNAs in the 3’UTR region. Using HOS cells, we co-transfected luciferase reporter vectors containing the 3’UTR sequence of these mRNAs as well as miR-4270 and miR-342 mimics or their corresponding hairpin inhibitors (**Figure 7**).

*In silico* analysis identified a potential binding site for miR-4270 between nucleotides 556-562 in BCL2-3’UTR (**Figure 7**). MiR-4270 did not significantly decrease (16%) the luciferase activity of the WT-BCL2-3’UTR vector compared with miR-Ctrl. Co-transfection of anti-miR-4270 led to a slight increase in the luciferase activity of the WT-BCL2-3’UTR (12% relative to miR-Ctrl, ns), in favor of a specific inhibitory effect of miR-4270. Nevertheless, due to the analyses of Bcl-2 protein expression and of the poor inhibitory effects on luciferase activity, we concluded that miR-4270 does not target directly *BCL2* mRNA in HOS cells. MiR-342 also had a potential binding site on the WT-BCL2-3’UTR vector and its transfection decreased Bcl-2 protein expression. Nevertheless, as previously described (27), it did not significantly decrease (22%) the luciferase activity of the WT-BCL2-3’UTR vector. Moreover, co-transfection with anti-miR-342-5p did not significantly reversed luciferase activity of the vector. As previously shown (27), despite its inhibitory effect on Bcl-2 protein expression, we cannot confirm that *BCL2* mRNA is a direct target of miR-342-5p in HOS cells.

We identified two presumptive binding sites for miR-4270 in BCL2L1-3’UTR at positions 326-333 and 738-744 (**Figure 7**). We have already demonstrated direct binding of miR-342-5p at position 679-686 and 1407-1413 of BCL2L1-3’UTR in HOS cells (27). As expected, miR-342-5p induced a great decrease in the luciferase activity of the WT-BCL2L1-3’UTR vector (70%, p<0.01) and anti-miR342-5p reversed this decrease. The co-transfection of miR-4270 resulted in a significant decrease (35%, p<0.05) in the luciferase activity of the reporter vector. The co-transfection of anti-miR-4270 restored the luciferase activity to the miR-Ctrl level, which is consistent with an inhibitory effect of miR-4270. Therefore, in HOS cells, miR-4270 downregulated Bcl-XL protein expression through direct binding to the 3’UTR of *BCL2L1* mRNA.

*In silico* analysis revealed that both miRNAs have a potential binding site in MCL1-3’UTR: between nucleotides 2427-2433 for miR-342-5p and between nucleotides 1585-1591 for miR-4270 (**Figure 7**). MiR-342-5p did not decrease the luciferase activity of the WT-MCL1-3’UTR vector, indicating no post-transcriptional regulation of *MCL1* mRNA by miR-342-5p, in accordance to the analysis of Mcl-1 protein expression (**Figure 6**). Surprisingly, as anti-miR-Ctrl, anti-miR-342-5p increased RLU beyond the miR-Ctrl level (80% relative to miR-Ctrl). MiR-4270 reduced the luciferase activity of the MCL1-3’UTR vector by 15% compared with miR-Ctrl, but this inhibitory effect was not statistically significant. Anti-miR-4270 had also an inhibitory effect, indicating a non-specific effect of miR-4270 on luciferase activity. Therefore, despite its inhibitory effect on Mcl-1 protein expression, we cannot confirm that *MCL1* mRNA is a direct target of miR-4270 in HOS cells.

## 4 Discussion

Osteosarcomas and chondrosarcomas are the most common forms of bone sarcomas. Today, osteosarcoma is a well-treated tumor given the efficacy of combination chemotherapy associated with surgery. However, for patients with metastases or recurrences, the survival rate is much lower. For chondrosarcomas, these tumors are resistant to conventional chemotherapy and radiotherapy and surgery remains the only effective therapy. Nevertheless, the location of the tumor may not allow surgery. The use of therapeutic miRNAs has the advantage of being a multi-target approach. Moreover, miRNAs may lead to the discovery of new therapeutic targets for clinical applications (14, 17, 30, 31).

Among miRNAs decreasing SW1353 cell proliferation in real-time cell analysis, we focused on four miRNAs potentially targeting the anti-apoptotic members of the Bcl-2 family. Indeed, Bcl-xL and Bcl-2 are promising targets in the treatment of chondrosarcomas and osteosarcomas (20–24). In a previous study, we demonstrated a suppressive role for miR-342-5p on chondrosarcoma cells through direct inhibition of Bcl-xL and Bcl-2 (26). We also showed that miR-491-5p is an apoptomiR in chondrosarcoma cells, through the direct inhibition of Bcl-xL and the repression of epidermal growth factor receptor (EGFR) expression. In a second study performed on osteosarcoma cells, we demonstrated a direct inhibition of Bcl-xL by both miRNAs, and their concomitant induction of apoptosis (27).

RTCA and endpoint morphological analysis of high-throughput screening of miRNAs on SW1353 cells were performed in glass culture plates under normoxia. However, chondrosarcomas are predominantly hypoxic tumors. We therefore carried out an individual functional validation of miR-16-1-3p, miR-646, miR-3667-3p and miR-4270 in conventional plastic culture plates, under normoxia and hypoxia. We were also interested in the chemosensitizing effects of these miRNAs. Unfortunately, we did not confirm the potential cytotoxic effect of the selected miRNAs on the SW1353 chondrosarcoma cell line. In addition, the tested miRNAs had no chemosensitizing effects in our experimental culture conditions, in contrast to what we observed in the high-throughput screening. However, we demonstrated the antiproliferative and cytotoxic effects of miR-4270 on the HOS and MG-63 osteosarcoma cell lines. We showed that miR-4270 had only a significant antiproliferative effect on the SaOS-2 osteosarcoma cell line. In addition, we were unable to demonstrate a potential chemosensitizing effect of these four miRNAs on any of the three osteosarcoma cell lines.

Human miR-16, also known as miR-16-5p, originates from two loci, *MIR-16-1* and *MIR-16-2*. Both loci produce the same guide strand miR-16-5p, but produce different passenger strands, miR-16-1-3p and miR-16-2-3p. Calin et al. found *MIR-16-1* locus deletion and/or miR-16 downregulation in the majority of chronic lymphocytic leukemia (CLL) cases (32). Since this study, miR-16 has been reported to be a tumor suppressor in many types of cancer, and as a potential oncomiR in some types of cancer (33). Nevertheless, miR-16-1-3p has been studied very little. Regarding osteosarcomas, the expression of miR-16-5p and miR-16-2-3p is downregulated in osteosarcoma samples in comparison to the healthy bone, and miR-16-1-3p expression is undetectable (34). Maximov et al. (2019) demonstrated the tumor suppressive properties of miR-16-1-3p, miR-16-2-3p and miR-16-5p *in vivo* and their capacity to reduce invasion *in vitro* (35). In our study, miR-16-1-3p induced a significant decrease in the viability and proliferation only on HOS cells, and we failed to demonstrate its cytotoxic or apoptotic effects 72h after transfection. In comparison with our study, Maximov et al. (2019) performed analysis of the induction of apoptosis later (5 days after lentiviral infection) (35). Furthermore, in contrast to our approach, they used stable clones overexpressing miRNAs because HOS polyclonal cultures lose overexpression of miRNAs too quick (35). Therefore, our experimental methods may not have been appropriate for miR-16-1-3p to induce cell death in osteosarcoma cell lines and in the SW1353 chondrosarcoma cell line.

MiR-646 is downregulated in many cancers and increasing evidence supports its tumor suppressive role (36). The function of miR-646 has been investigated in studies performed on osteosarcomas, but none on chondrosarcomas. Two studies showed that miR-646 inhibits migration and invasion in the U2OS cell line (37, 38). Another study showed that miR-646 is downregulated in four osteosarcoma cell lines (MG-63, SaOS-2, HOS and U2OS) and in osteosarcoma tissues (39). Moreover, low expression of miR-646 is associated with metastasis. They showed that the transfection of miR-646 in MG-63 cells inhibited cell proliferation, migration and invasion, but they did not investigate the induction of cell death. They concluded that miR-646 may be a tumor suppressor in osteosarcoma *via* its direct target of Fibroblast Growth Factor 2 (FGF2). In our study, miR-646 was also able to significantly reduce the viability and proliferation of HOS, MG-63 and SaOS-2 cells, but it was not able to induce cytotoxicity in all these cell lines. Because it did not induce cell death in the HOS and SW1353 cell lines, we did not continue our investigations in the other cell lines, nor did we study its anti-metastatic capacities. Further investigations may lead to the same conclusions as the Sun et al. study (39), but we focused on the more promising miR-4270 on osteosarcoma cells.

For miR-3667-3p, to our knowledge, only one other study has addressed its role in cancer. MiR-3667-3p targets the lncRNA (long non-coding RNA) PCAT-1 (Prostate cancer associated transcript-1) in prostate cancer (40). PCAT-1 promotes prostate cancer cell proliferation through positive post-transcriptional regulation of c-Myc. MiR-3667-3p would therefore have anti-suppressor properties, but in our study, despite antiproliferative effects, we did not confirm its potential cytotoxic effect, on any of the cell lines studied.

MiR-4270 seems to play a dual role in cancer. It is downregulated in hepatocellular carcinoma (HCC) tissues and gastric cancer tissues (41–43). It inhibits cancer cell proliferation and metastasis in HCC by directly targeting stabilin-2 (STAB2) (42). Circ_0005556, a circular RNA, sponged miR-4270, resulting in MMP19 overexpression, and subsequent proliferation, migration and invasion of gastric cancer cells (43). Intriguingly, other studies have shown that circulating miR-4270 displays upregulated levels in metastatic gastric cancer and breast cancer patients, with the potential use of miR-4270 as a diagnostic or prognostic biomarker (44, 45). Hao et al. reported that the levels of miR-4270 modulate the irradiation-sensitivity of nasopharyngeal carcinoma cells through modulation of p53 *in vivo* (46). Collectively, these results suggest that different levels of miR-4270 expression may be associated with different cancers and that miR-4270 likely has different functions.

In our study, the best antiproliferative effect of miR-4270 was observed on SW1353 chondrosarcoma cells 96 h post-transfection in the high-throughput screening, but we failed to validate its cytotoxic effect in the functional analysis 72 h post-transfection. A longer incubation period may be necessary to validate the effects obtained during the screening. However, functional analyses revealed antiproliferative and cytotoxic effects of miR-4270 on the HOS and MG-63 osteosarcoma cell lines 72 h post-transfection, but only antiproliferative effects on the SaOS-2 osteosarcoma cell line. We did not detect any chemosensitizing effect of this miRNA in the presence of CDDP in any cell line in our experimental conditions. Sublethal doses of CDDP may not be adequate to reveal the chemosensitizing effects of miR-4270 on osteosarcoma cells. In the HOS and MG-63 cell lines, we clearly observed an increase in cleaved PARP and cleaved caspase-3 under miR-4270 treatment. In addition, as shown for miR-342-5p (27), miR-4270 increased caspase-3/7 activity in both cell lines, whereas we detected only PARP cleavage in SaOS-2 cells. In our previous study, we demonstrated that miR-342-5p induces cell death in SaOS-2 cells, without inducing caspase-3 cleavage and caspase-3/7 activity, but we detected the induction of cleaved PARP (27). An annexin V/PI assay could help to corroborate these results, but miR-4270 seemed at least effective in inducing apoptosis in HOS and MG-63 cells.

*In silico* analysis showed that the anti-apoptotic proteins Bcl-2, Bcl-xL, and Mcl-1 are potential targets of miR-4270. Bcl-2 protein expression was significantly decreased by miR-342-5p, but not by miR-4270 in the three osteosarcoma cell lines. However, we did not validate Bcl-2 as a direct target of either miRNAs in HOS cells, as previously described for miR-342-5p (27). We demonstrated that Bcl-xL protein expression is significantly reduced by miR-4270 in HOS and MG-63 cells and to a lesser extent in SaOS-2 cells. We demonstrated for the first time, that Bcl-xL is a direct target of miR-4270. We also demonstrated that miR-4270 decreased Mcl-1 protein expression particularly in MG-63 and SaOS-2 cells. However, we did not confirm that Mcl-1 is a direct target of miR-4270 in HOS cells. Furthermore, miR-4270 was able to increase the expression of the pro-apoptotic protein Bak especially in the HOS and MG-63 cell lines. To gain insights into the mechanism of action of miR-4270, we could use selective inhibitors of Bcl-xL (WEHI-539, A-1331852) or siRNA against Bcl-xL to compare their effects to that of miR-4270.

We used three osteosarcoma cell lines from young Caucasian patients. Cell lines did not have the same aggressiveness. HOS cell line had high aggressiveness compared to SaOS-2 and MG-63 cell lines, and MG-63 cells had less invasive/migratory capacities than SaOS-2 cells (47). Genomic analyses revealed key differences in the three osteosarcoma cell lines used. Deletion and rearrangement of the p53 tumor suppressor gene were found in MG-63 and SaoS2 cell lines, with no p53 expression detected. A point mutation within the p53 coding sequence has been described in HOS cells which results in overproduction of mutant p53 (48). Furthermore, several mRNAs and miRNAs were differentially expressed in highly aggressive osteosarcoma cell lines compared with non-aggressive cell lines. Nevertheless, miR-4270 was not identified in the list of miRNAs significantly upregulated or downregulated in the aggressive cell lines (47). Further investigations could be needed to link the differential effect of miR-4270 to the genetic background of the 3 cell lines.

Numerous signaling pathways have been linked to tumor progression in osteosarcoma. Wnt/β-catenin signaling has been shown to be oncogenic in a wide range of tumor types, including osteosarcoma (49). NF-κB appears to play a causative role in the malignancy of osteosarcoma (50). Moreover, crosstalk between NF-κB and Wnt/β-catenin pathways link inflammation with tumorigenesis (51). Overexpression and abnormal activation of Raf/MEK/ERK signaling pathway may regulate tumor proliferation, migration, and metastasis in osteosarcoma as well as in other malignancies (52). PI3K/Akt is a critical driver of oncogenesis in OS and largely studied with the development of inhibitors of mTOR, the most important downstream molecule of the PI3K/AKT pathway (53). To the best of our knowledge, no study has explored the effect of miR-4270 on these signaling pathways. This could be explored in the future. However, our preliminary data suggested a possible downstream inhibition of the AKT and ERK signaling pathways on MG-63 cells (data not shown).

Overall, we demonstrated the tumor suppressive role of miR-4270 on HOS and MG-63 osteosarcoma cell lines. MiR-4270 had antiproliferative effects and some anti-apoptotic effects on SaOS-2 cell line, but these latter effects were not sufficient to induce cell death in this osteosarcoma cell line. Likewise, miR-4270 did not induce cell death in the SW1353 chondrosarcoma cell line, despite its antiproliferative effects. Moreover, we showed that miR-4270 does not affect healthy primary human articular chondrocytes (HAC) cell cycle, nor does it cause any cell death (**Figure S8**). As for miR-342-5p (26), these two experiments suggest its biosafety on non‐cancerous cells. However further investigations would be required to verify tumor suppressive role of miR-4270 in *in vivo* studies or in three-dimensional (3D) OS cell culture models. Nevertheless, miR-4270 therefore appears to have a specific function according to the type of tumor, and sometimes it can act as an oncogene or as a tumor suppressor as described above. Furthermore, it may have a more or less pronounced effect depending on the cell line used, particularly according to its altered status of differentiation.

## 5 Conclusions

Our study demonstrated antiproliferative and cytotoxic effects of miR-4270 on HOS and MG-63 osteosarcoma cell lines. MiR-4270 also significantly induced apoptosis in these cell lines. Here, for the first time, Bcl-xL was identified as a direct target of miR-4270. MiR-4270 also decreased Mcl-1 protein expression, whereas it increased that of Bak. We already demonstrated the therapeutic potential of specifically targeting Bcl-xL and Bcl-2 on chondrosarcoma and osteosarcoma cells. Here, we provide additional evidence that Bcl-xL could be used as a relevant therapeutic target in combination with chemotherapy in osteosarcomas, with BH3-mimetic derivates for example to inhibit Bcl-xL family members and improve sensitivity to conventional chemotherapy.

## Data availability statement

The original contributions presented in the study are included in the article/**Supplementary Material**. Further inquiries can be directed to the corresponding author.

## Ethics statement

The study was performed in full accordance with local ethics committee guidelines and all the cartilage samples were collected after written and informed consent of the donors according to French legislation. All the experimental protocols were approved by the Agence de la Biomédecine (research protocol n° PFS21-025) and French Ministry of Higher Education and Research (Ethics Committee for Research on Human Samples CODECOH: DC 2014–2325).

## Author contributions

Conceptualization: CV, AB, CD, LP, NG, FL, PG. Methodology: CV, AB, EB, FR, FL, PG. Formal analysis: CV, FB, MJ, AB, EB, NG, FL, PG. Software: AB, EB. Validation: CV, FL, PG. Investigation: CV, FB, MJ, EB, FL. Resources: CD, LP, FR, NG, PG. Data Curation: EB, CD. Writing - Original Draft Preparation: CV, FL. Writing - Review & Editing: FL, PG. Visualization: CV, MJ, CD, NG, FL, PG. Supervision: FL, PG. Project Administration: FL, PG. Funding Acquisition: CD, LP, FL, PG. All authors read and approved the final manuscript.
