# Supplementary material for: MiR-4270 acts as a tumor suppressor by directly targeting Bcl-xL in human osteosarcoma cells
Source: Front Oncol. 2023 Aug 31;13:1220459. doi: 10.3389/fonc.2023.1220459 (PMC10501397; doi:10.3389/fonc.2023.1220459)
Supplement: Supplementary file 1 [file DataSheet_1.pdf]

Supplementary Material

MiR-4270 acts as a tumor suppressor by directly targeting Bcl-xL in human osteosarcoma cells

Clément Veys[1], Flavie Boulouard[1,2], Abderrahim Benmoussa[1,3], Manon Jammes[1], Emilie Brotin[4,5,6], Françoise Rédini[7], Laurent Poulain[5,6], Nicolas Gruchy[1,2], Christophe Denoyelle[4,5,6], Florence Legendre[1†] and Philippe Galera[1†*]

† These authors contributed equally to this work and share the last authorship
* Correspondence: Philippe Galéra ; philippe.galera@unicaen.fr

SW1353

A.

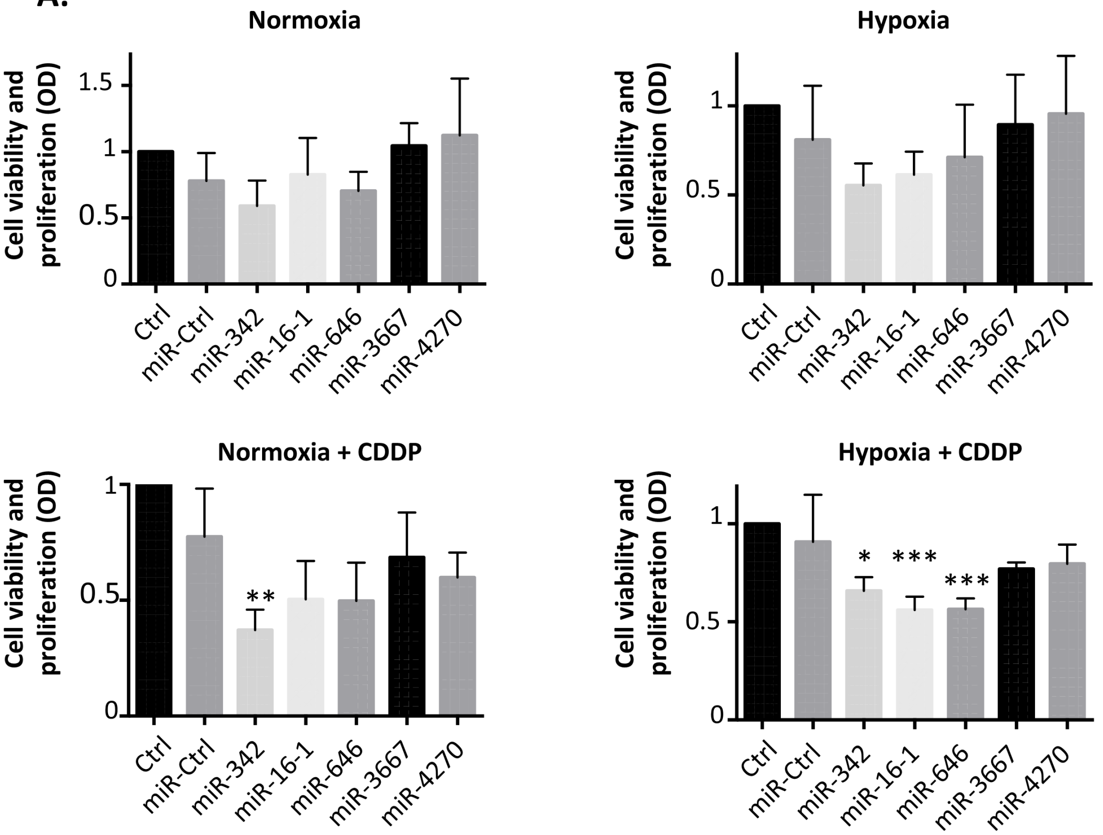
Normoxia
Hypoxia
Normoxia + CDDP
Hypoxia + CDDP
Cell viability and proliferation (OD)
0
0.5
1
1.5
**
*
***
***
Ctrl
miR-Ctrl
miR-342
miR-16-1
miR-646
miR-3667
miR-4270

B.

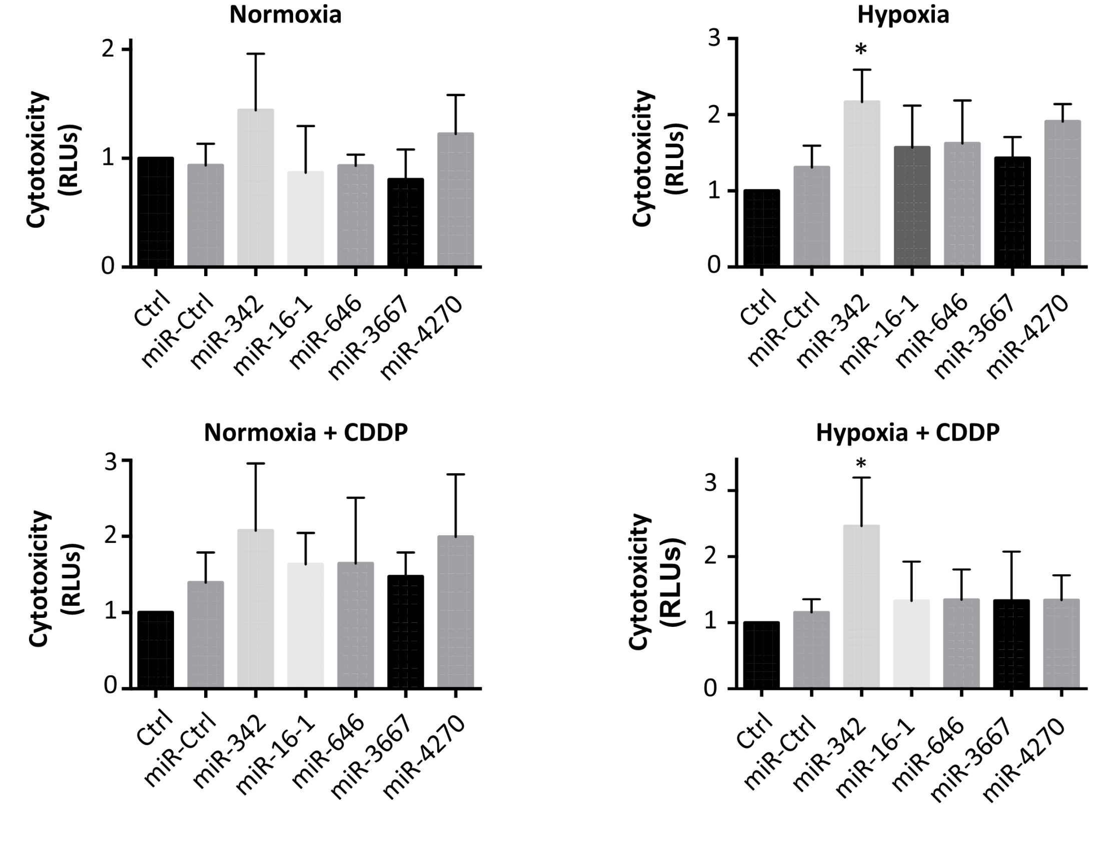
Normoxia
Hypoxia
Normoxia + CDDP
Hypoxia + CDDP
Cytotoxicity (RLUs)
0
1
2
3
*
*
Ctrl
miR-Ctrl
miR-342
miR-16-1
miR-646
miR-3667
miR-4270

Supplementary figure S1: Analysis of miRNA-induced cytotoxicity and impact of miRNAs on the viability and proliferation **of SW1353 cells.** SW1353 cells, cultured under normoxia or hypoxia, were transfected with miRNAs 24 h after seeding. Cells were treated with CDDP 48 h post-transfection for 24 h. Analyses were then carried out 72 h post-transfection. (A) Cell viability and proliferation were evaluated and expressed as the mean OD ± SD of four independent experiments. (B) The cytotoxicity of the miRNAs was evaluated and expressed as the mean RLU ± SD of four independent experiments. The significance of the results between miR-Ctrl and miRNA-treated cells was assessed using one-way ANOVA (*: $p<0.05$, **: $p<0.01$, ***: $p<0.001$).

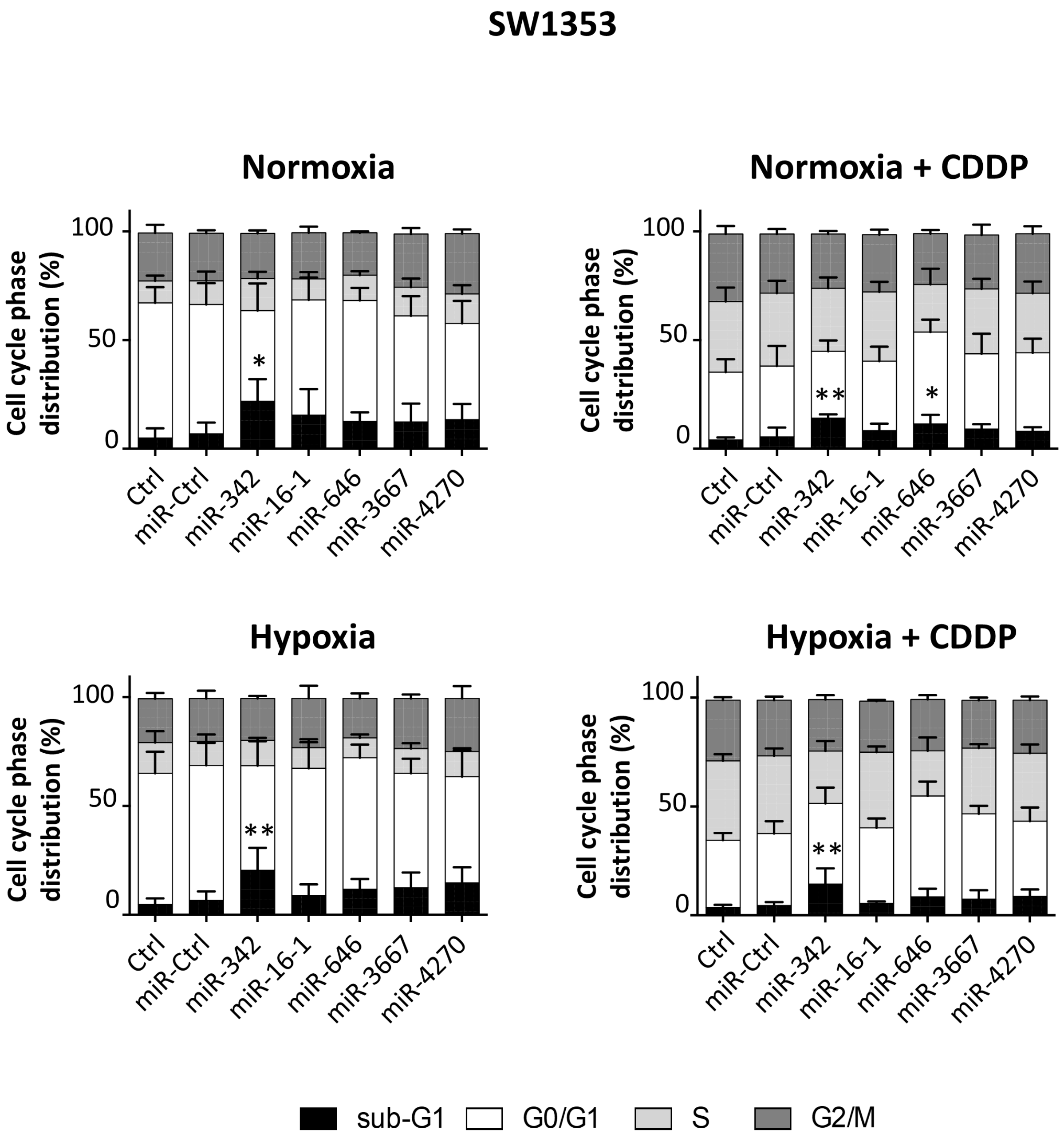

Supplementary figure S2: Analysis of cytotoxic or chemosensitizing effects of miRNAs on the SW1353 chondrosarcoma cell line. SW1353 cells, cultured under normoxia or hypoxia, were transfected with miRNAs 24 h after seeding. Cells were treated with CDDP 48 h post-transfection for 24 h. Cell cycle analysis was realized 72 h post-transfection. The histograms represent the analysis of at least four independent experiments

(mean ± SD). The significance of sub-G1 events between miR-Ctrl and miRNA-treated cells was evaluated using one-way ANOVA (*: $p<0.05$, **: $p<0.01$).

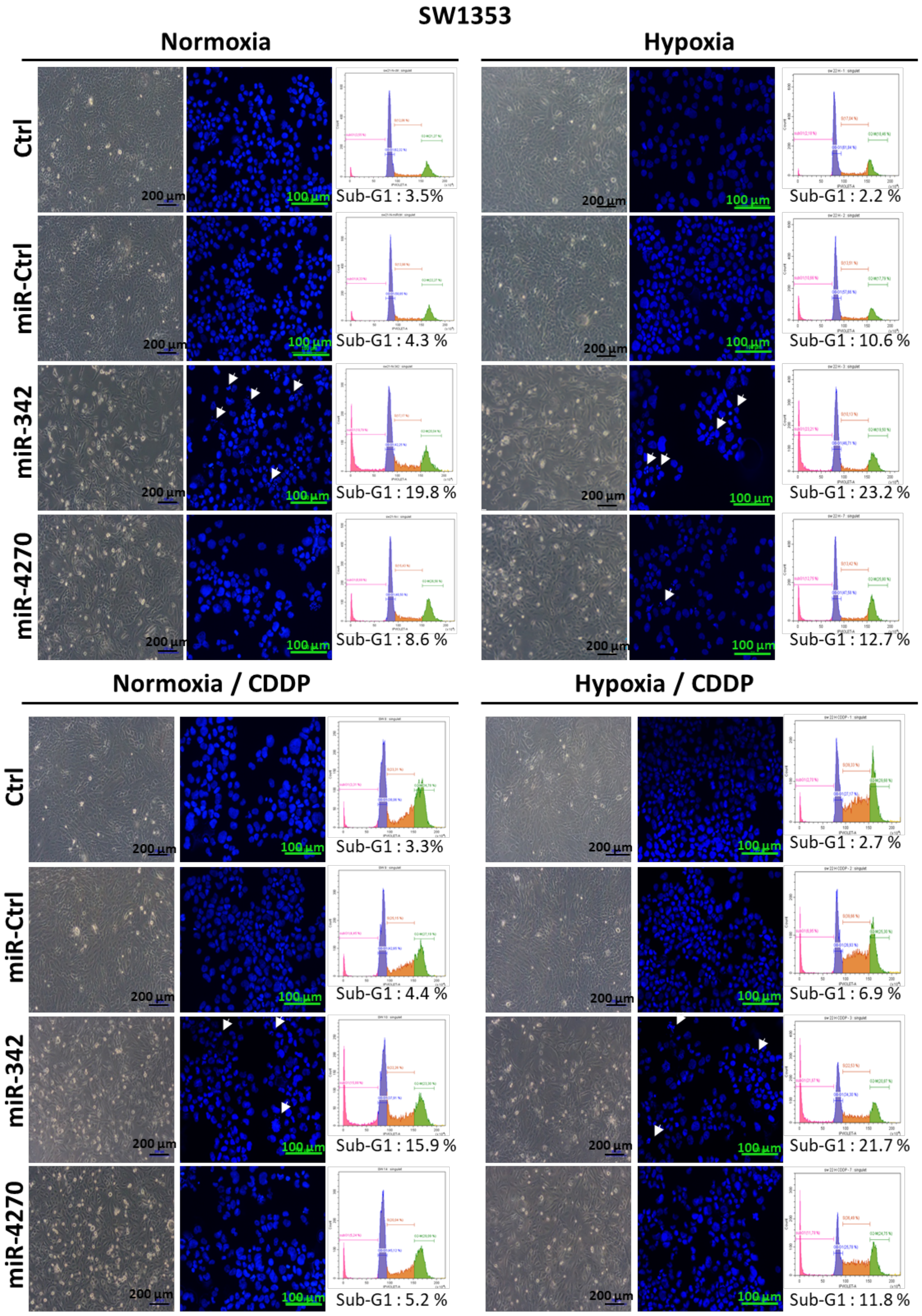

Supplementary figure S3: Characterization of the effects of miR-342 and miR-4270 on SW1353 cells. SW1353 cells, cultured under normoxia or hypoxia, were transfected with miRNAs 24 h after seeding. Cells

were treated with CDDP 48 h post-transfection for 24 h. Analyses were carried out 72 h post-transfection. In the left-hand panels, cell morphology was analyzed at the end of the experiment. In the middle part of each panel, nuclear morphology was analyzed after DAPI staining. White arrowheads show cellular debris and cells with condensed and/or fragmented chromatin. In the right-hand panels, DNA content histograms were obtained by flow cytometry. Images shown are representative of at least four independent experiments.

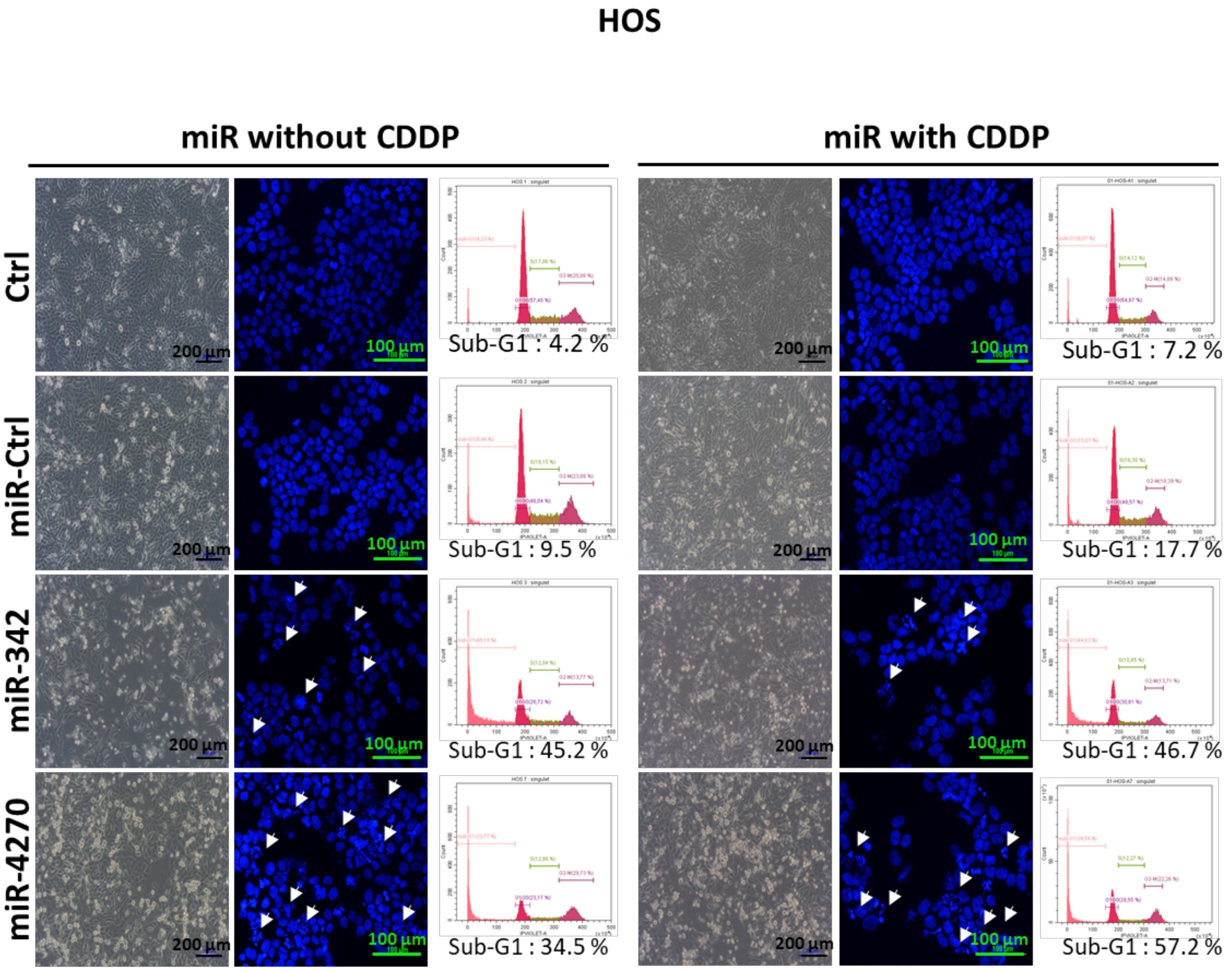

Supplementary figure S4: Characterization of the effects of miR-342 and miR-4270 on HOS cells. HOS cells were transfected with miRNAs 24 h after seeding as described in Figure 4A. Analyses were carried out 72 h post-transfection. In the left-hand panels, cell morphology was analyzed at the end of the experiment. In the middle panels, nuclear morphology was analyzed after DAPI staining. White arrowheads show cellular debris and cells with condensed and/or fragmented chromatin. In the right-hand panels, DNA content histograms were obtained using flow cytometry. Images shown are representative of four independent experiments.

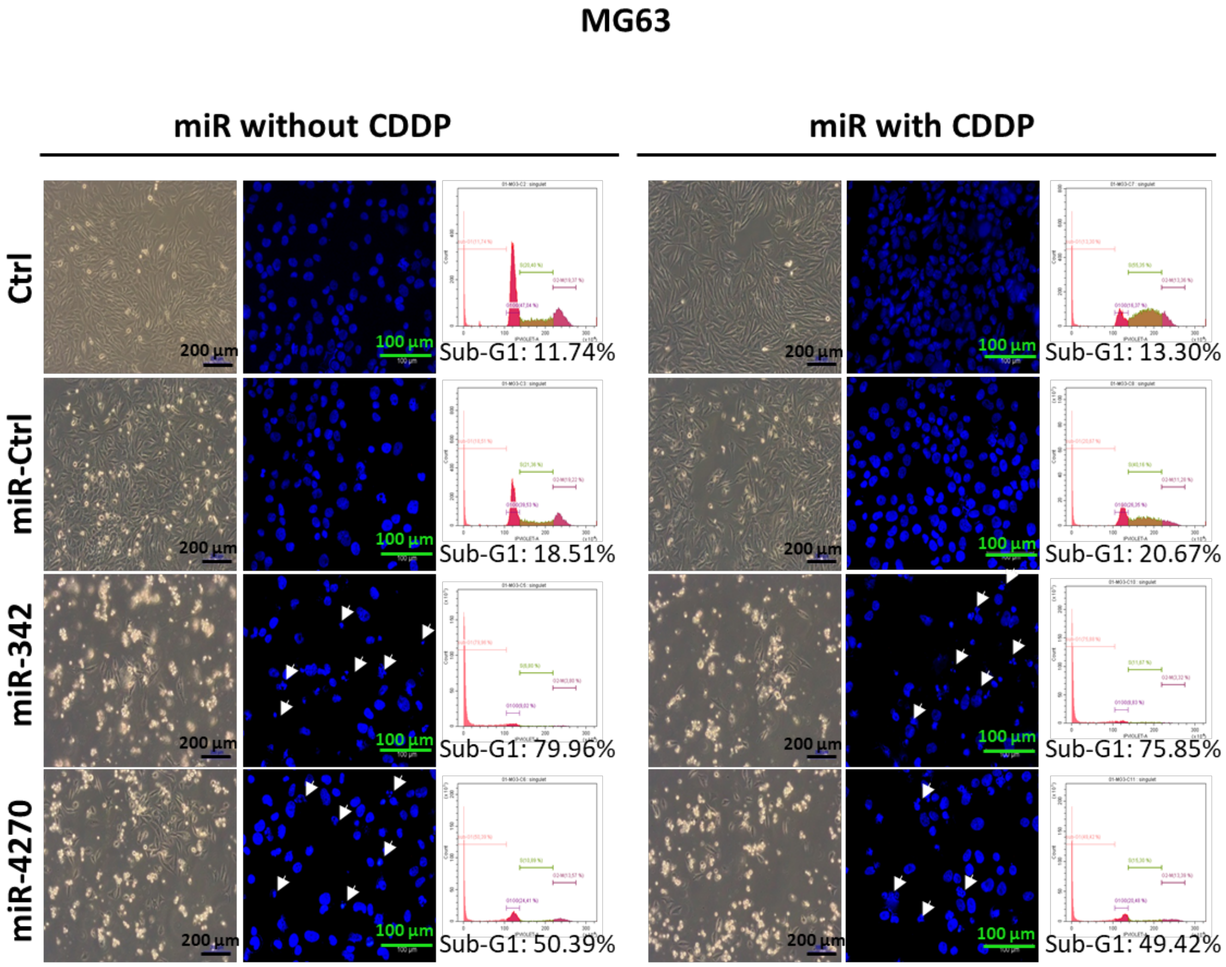

Supplementary figure S5: Characterization of the effects of miR-342 and miR-4270 on MG-63 cells. MG-63 cells were transfected with miRNAs 24 h after seeding as described in Figure 4B. Analyses were carried out 72 h post-transfection. In the left-hand panels, cell morphology was analyzed at the end of the experiment. In the middle panels, nuclear morphology was analyzed after DAPI staining. White arrowheads show cellular debris and cells with condensed and/or fragmented chromatin. In the right-hand panels, DNA content histograms were obtained using flow cytometry. Images shown are representative of independent experiments.

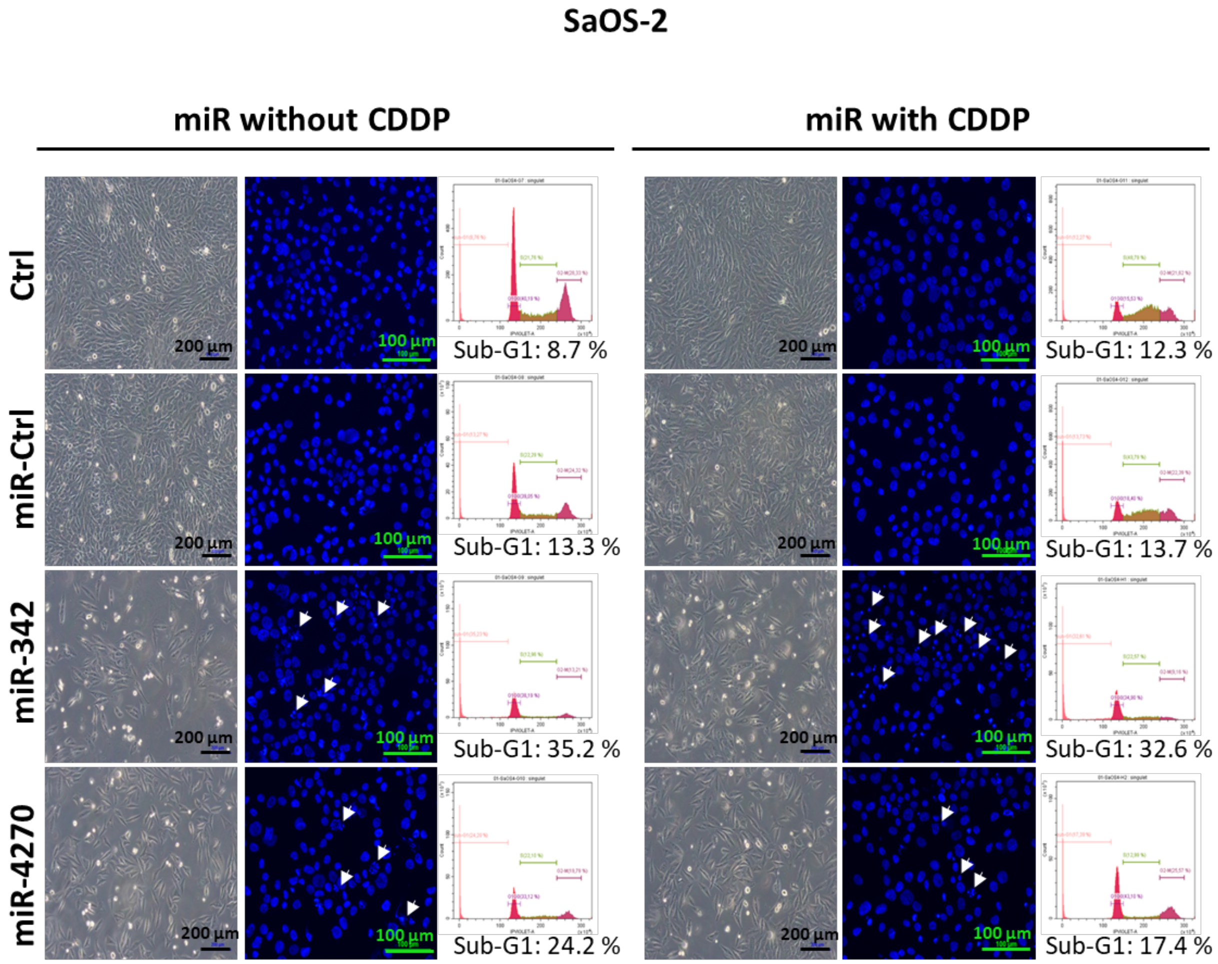

Supplementary figure S6: Characterization of the effects of miR-342 and miR-4270 on SaOS-2 cells. SaOS-2 cells were transfected with miRNAs 24 h after seeding as described in Figure 4C. Analyses were carried out 72 h post-transfection. In the left-hand panels, cell morphology was analyzed at the end of the experiment. In the middle panels, nuclear morphology was analyzed after DAPI staining. White arrowheads show cellular debris and cells with condensed and/or fragmented chromatin. In the right-hand panels, DNA content histograms were obtained by flow cytometry. Images shown are representative of four independent experiments.

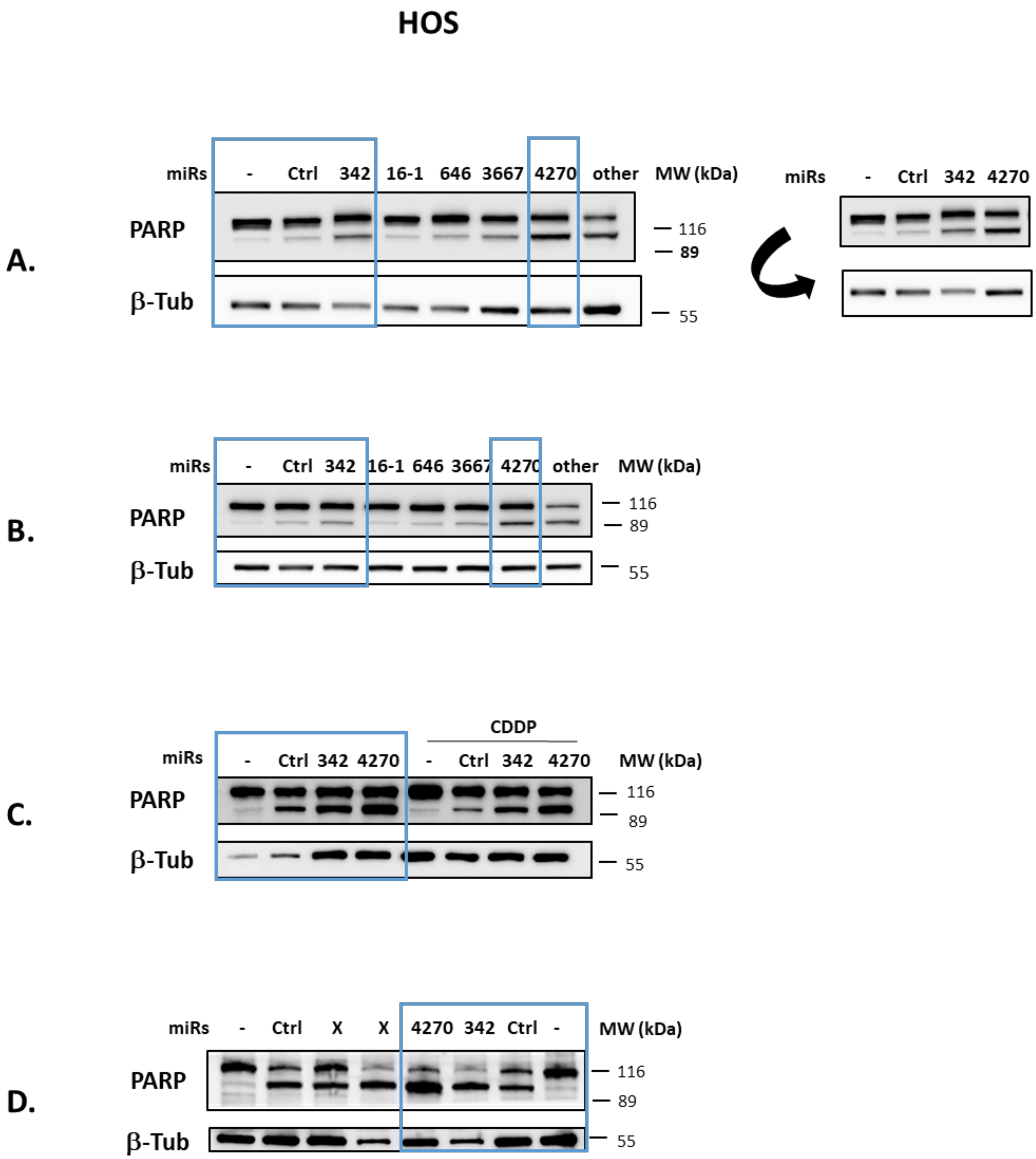

Supplementary figure S7: Analysis of the apoptotic effects of miR-342-5p and miR-4270 on HOS cell line. HOS cells were transfected with miRNAs 24 h after seeding as described in Figure 5A. PARP levels were analyzed 72 h post-transfection. Blots of four independent experiments are shown.

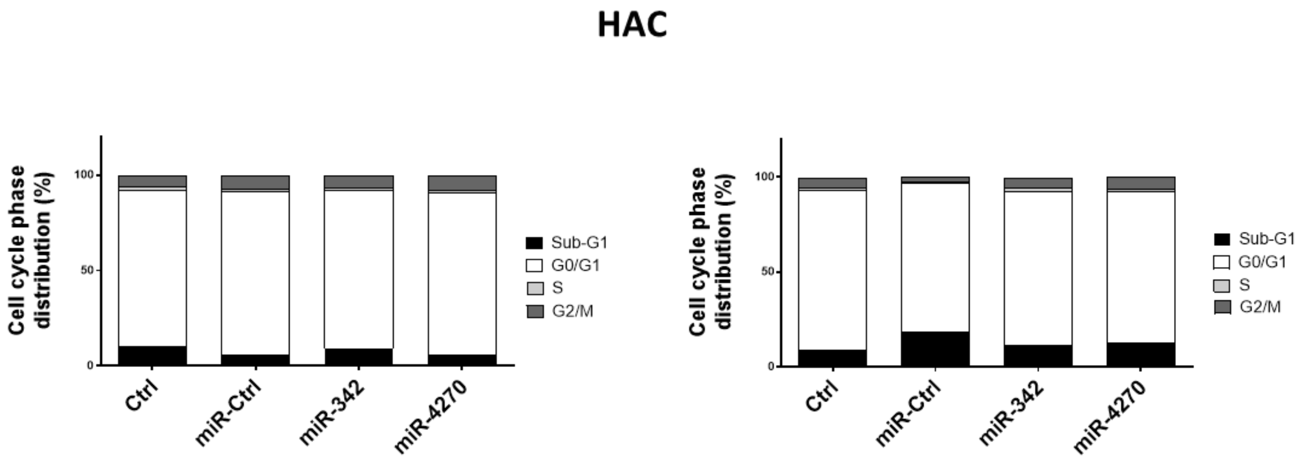

Supplementary figure S8: Analysis of the cytotoxic effects of miR-342 and miR-4270 on healthy HAC cultured under normoxia. Primary HAC were cultured under normoxia. They were transfected with 20 nM miRNAs 5 days after seeding. DNA content histograms of two independent experiments were obtained by flow cytometry 72h post-transfection.
